# Origin of the Temperature-Induced Gap Bowing of Formamidinium–Methylammonium Lead Iodide Perovskites: Role of Cationic Rattlers

**DOI:** 10.1021/acs.jpclett.6c01965

**Published:** 2026-07-28

**Authors:** Kai Xu, Adrián Francisco-López, Bethan L. Charles, M. Isabel Alonso, Miquel Garriga, Mark T. Weller, Alejandro R. Goñi

**Affiliations:** † 54449 Institut de Ciència de Materials de Barcelona (ICMAB-CSIC), Carrer dels Til.lers, 08193 Cerdanyola del Vallès, Spain; ‡ Department of Chemistry & Centre for Sustainable Chemical Technologies, University of Bath, Claverton Down, Bath BA2 7AY, U.K.; ¶ School of Electrical, Electronic and Mechanical Engineering, Queens Building, 1980 University of Bristol, Bristol BS8 1TR, U.K.; § Department of Chemistry, 2112 Cardiff University, Wales CF10 3AT, U.K.; ∥ ICREA, Passeig Lluís Companys 23, 08010 Barcelona, Spain

## Abstract

Understanding the temperature dependence of semiconductor band gaps is essential for optimizing optoelectronic devices. However, the origin of the pronounced temperature-induced gap bowing observed in low-temperature phases of formamidinium–methylammonium (FA-MA) lead iodide perovskites has remained elusive until now. By combining temperature and pressure-dependent photoluminescence measurements on FA_*x*_MA_1–*x*_PbI_3_ single crystals with *x* ∈ [0, 1], we unravel the origin of this bowing. Both thermal expansion and electron–phonon interaction effects are responsible. The latter is the leading term, driven by activation of an anomalous electron–phonon coupling mechanism linked to mixed vibrational modes, which combine inorganic-cage phonons involving octahedral tilting with low-frequency FA librations, called FA *rattlers*. As shown in the revised composition–temperature phase diagram, this occurs in the orthorhombic and (pseudo)­tetragonal low-temperature phases, presumably featuring stripe domains with alternating octahedral tilt-axis patterns for *x* between 0.2 and 0.9. This sheds light on an intriguing behavior of lead halide perovskites that directly affects their optoelectronic properties.

## Graphical abstract

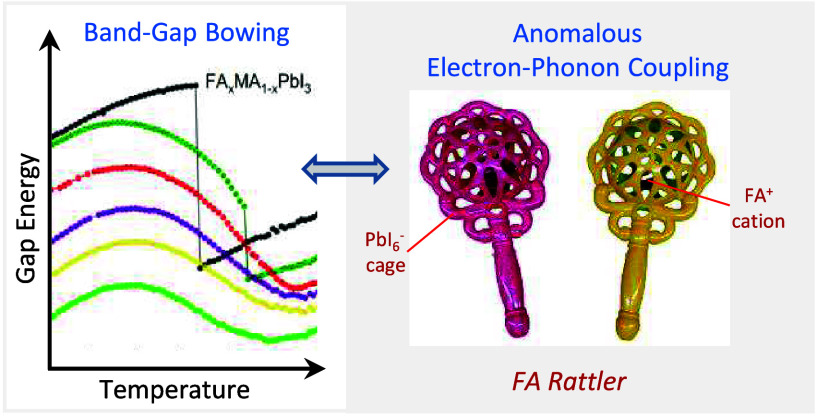

Semiconductors are characterized by an energy band gap typically located in the visible spectral region, from the near-infrared to the near-ultraviolet. Therefore, a fundamental understanding of what determines the semiconductor band gap and its temperature dependence is essential for the design and performance optimization of optoelectronic devices. For instance, the gap determines both the efficiency of a solar cell^1^ and the color of a light-emitting device.^2^ Generally speaking, the effects of temperature on the band structure of any semiconductor are described by two terms related to thermal expansion (TE) and electron–phonon (EP) interaction.^3–5^ The former arises from the intrinsic anharmonicity of the crystal potential, which leads to expansion or contraction of the crystal lattice when temperature is raised or lowered, respectively. The gap thus partly changes due to the temperature-induced volume variations. It is important to note that the TE term can be experimentally assessed by determining the gap pressure coefficient in high-pressure experiments.^6^ In contrast, the energy renormalization of the electronic states produced by lattice vibrations is essentially due to the smearing of the crystal potential and the scattering of electrons by (virtual) phonons. Both effects are proportional to the Bose–Einstein phonon occupation number, hence the EP term.^7^ At a given temperature, the magnitude and sign of the EP term can be directly obtained from the difference of two experimentally determined quantities: the measured temperature slope of the gap and the TE term derived from the measured gap pressure coefficient.^8^ However, it is a common practice to describe the EP term using the Einstein-oscillator model,^9–11^ which considers an effective electron–phonon coupling constant for phonons with an average frequency derived from peaks in the phonon density of states. For a discussion of the current status and challenges in accounting for the electron–phonon renormalization in electronic structure calculations we refer to a recent perspective work.^12^

Metal halide perovskites (MHPs) with formula AMX_3_, with divalent Pb or Sn in the metal M-site, Cs, methylammonium (MA) or formamidinium (FA) as the A-site cation, and X as a halogen atom (Cl, Br or I), exhibit around room temperature a linear dependence on temperature of the gap with positive slope. This was obtained after comparing the near-ambient temperature gap slope of ca. 20 MHPs in nanocrystalline, thin film or bulk form (see ref 13 and references therein). For MAPbI_3_ it has been shown that this positive temperature gap slope picks up similarly strong contributions from thermal expansion and electron–phonon interaction effects.^14^ However, a recent breakthrough in electron–phonon calculations in locally disordered (polymorphous) MHPs^15^ indicates that the relative weight of EP term can vary from 27% to 97% with increasing local disorder, going from FA, to MA and to Cs-containing perovskites.^16,17^ In any case, for the correct account of the gap temperature dependence the TE term cannot be neglected, even less in the low-temperature phases, for which the gap pressure coefficient is strongly temperature dependent, as shown for MAPbI_3_ ^18^ and MAPbBr_3_ ^19^ and will be shown here for the FA_*x*_MA_1–*x*_PbI_3_ system. The calculations within the polymorphous model reproduce very well the sign and magnitude of the linear temperature dependence observed experimentally at around room temperature and above for more than 12 MHP compounds.^17^ A notable exception to the *linearity* rule is CsPbCl_3_, which exhibits a sign reversal in the temperature slope; i.e., the gap decreases with increasing temperature near ambient, both for nanocrystals^20^ and for thin films.^21^ As recently reported elsewhere,^22^ we demonstrated that only the electron–phonon interaction is responsible for the sign reversal, which occurs due to the activation of an anomalous electron–phonon coupling mechanism linked to vibrational modes characterized by synchronous octahedral tilting and the motion of the Cs cations inside the cage voids, the so-called Cs rattlers. These modes have been also invoked to understand the extremely low thermal conductivity of CsPbBr_3_.^23^ Another exception is the pronounced temperature-induced gap bowing exhibited by FA/MA mixed-cation lead iodide perovskites in low-temperature phases below ca. 250 K,^24,25^ the origin of which has remained elusive until now. In this letter we will show that the only way to account for this bowing is to introduce an additional Einstein oscillator in the EP term with negative amplitude, associated with an anomalous EP coupling term linked to FA-rattler modes instead of Cs. This occurs mainly in a low-temperature (pseudo)­tetragonal phase, presumably featuring stripe domains with alternating (90°) octahedral tilt-axis patterns for FA concentrations between 20% and 90%.^26^

Given the importance for MHPs of the unique interaction between crystal structure and A-site cation dynamics for their electronic (in particular, the gap) and vibrational (phonon spectrum) properties, it will be instructive to review the composition–temperature phase diagram of the FA_*x*_MA_1–*x*_PbI_3_ system to find a suitable explanation for what might be triggering the aforementioned anomalous EP coupling with the FA rattlers. For a general but complete account of the relationship between crystal structure and cation dynamics in MHPs, we refer to the work of Simenas et al.^27^ Different parts of the FA_*x*_MA_1–*x*_PbI_3_ phase diagram have been addressed by several authors.^25,26,28–31^ The relationship between structure and A-site cation dynamics has been revisited from two antagonist points of view regarding the interaction mechanism, that is, hydrogen bonding^32,33^ and dynamic steric interaction.^34^ The influence of the latter has been also considered to understand phonon interactions in MHPs as revealed by Raman scattering.^35^ Of particular interest for this work is the very recent molecular dynamics (MD) study of the phase diagram of FA_*x*_MA_1–*x*_PbI_3_, linking structure, dynamics and electronic band structure.^26^ The main result is the identification of a morphotropic phase boundary (MPB) for FA contents of ca. 27%, delineating the transition from the tetragonal phase with the *I*4/*mcm* space group^36^ and (in Glazer notation) out-of-phase *a* ^0^ *a* ^0^ *c* ^–^ octahedral tilt pattern, characteristic of the MA-rich lead iodide compounds, to the also tetragonal phase with *P*4/*mbm* symmetry^28,29^ but in-phase *a* ^0^ *a* ^0^ *c* ^+^ tilt pattern, typical of solid solutions with high FA content. Interestingly, this transition is accompanied by the formation of stripe domain structures with alternating tilt-axis patterns and, most importantly, the enhancement of dynamic disorder and electron–phonon coupling.^26^ Here we would like to propose that the (pseudo)­tetragonal phase stabilized below approximately 250 K and at intermediate FA concentrations, called the Tetra-III phase,^25^ has a layered character exhibiting a mosaic-like structure, where nanoscale regions with an alternating tilt pattern coexist within each stripe domain. In fact, for this phase the space group assignment from neutron scattering^29^ is uncertain, *P*4*bm* only being plausible, but indicating a certain degree of structural disorder present. This is the phase for which mainly, but not exclusively, the gap temperature dependence displays the pronounced bowing.

High-quality single crystals of FA_*x*_MA_1–*x*_PbI_3_ across the full composition range were synthesized using the inverse solubility method of Saidaminov et al.,^38^ as explained in Note #0 of the Supporting Information. The photoluminescence (PL) spectra were excited with the 633 nm line of a He–Ne laser using a very low incident light power below 2 μW (power density <15 W/cm^2^) to avoid any photodegradation of the samples.^39^ PL spectra were corrected for the spectral response of the spectrometer by normalizing each spectrum using the detector and the 600 grooves/mm grating characteristics. Temperature-dependent PL measurements were carried out by decreasing the temperature between ca. 365 and 6 K in steps of 5 K, using liquid helium in a gas flow cryostat from CryoVac. Room temperature high-pressure PL measurements were performed employing a gasketed diamond anvil cell (DAC). Anhydrous propanol was used as pressure transmitting medium at room temperature, which ensures good hydrostatic conditions and proved chemically inert to (FA,MA)­PbI_3_. In contrast, for the high-pressure PL measurements at low temperatures down to 4 K a specially designed He-bath cryostat (Konti-IT from Cryovac) was used that can allocate the DAC in its cold bore.^6^ For cryogenic high-pressure measurements, helium is the best possible pressure medium. The difficulty is that loading ought to be performed with the DAC immersed in superfluid helium, as explained in detail in the Note #1 of the Supporting Information.

Figure 1 panels a–c display the temperature evolution from ca. 6 to 365 K of the PL spectra of FA_*x*_MA_1–*x*_PbI_3_ single crystals for three compositions selected to have different crystal phases at low temperatures (see discussion below), namely, *x* = 0.2, 0.5, and 1.0, respectively. The spectral cascade for the rest of FA compositions were published elsewhere.^25^ All spectra were normalized to the absolute maximum intensity and vertically offset for clarity. To analyze the PL spectra, we used a Gaussian–Lorentzian cross-product function for describing the main peak which is ascribed to free-exciton recombination.^40^ The procedure for the line-shape analysis of the PL spectra is described in Note #2 of the Supporting Information. The values of the PL peak maximum obtained from a line-shape analysis of the PL spectra fits are plotted as a function of temperature in Figure 2 for the 11 compositions of the FA_*x*_MA_1–*x*_PbI_3_ series. For practical purposes we consider the shift of the PL peak energy with temperature to be representative of the shift of the gap.^8,41^ The sets of data points exhibit a clear decreasing trend of the gap at room temperature with increasing FA content, except for the two compositions *x* = 0.7 and 1 (open symbols in Figure 2). In the case of 70% FA content, a combined spectroscopic ellipsometry and temperature-dependent PL study^42^ indicates that this sample consists of FA- and MA-rich segregated regions separated by areas with high concentration of vacancies (predominantly of MA^43–45^ ), although maintaining an average stoichiometry of *x* = 0.7. Evidence of this is provided by the observation, in the low-temperature PL spectra, of two distinct, sharp lines with varying intensity, depending on the laser focus point (Figure 6 of ref 42). At high temperatures, these lines evolved into a single, broad PL peak but centered at slightly different energies. This clearly indicates the existence of compositional inhomogeneity at macroscopic distances greater than the laser spot (ca. 4 μm in diameter). For FAPbI_3_, in contrast, we were not able to study the pristine as-grown sample. For technical reasons, the sample had already transformed into the yellow hexagonal δ-phase when received. Although we were able to transform the FAPbI_3_ crystal back to the perovskite α-phase by heating it on a hot plate to about 80 °C for a few minutes,^46^ it seems that this treatment was somewhat detrimental for its crystal quality. However, in both cases, the sample inhomogeneity appears to have affected only the absolute value of the energy gap, but not its temperature dependence. This is evidenced by the fact that the first derivative curves align perfectly with the overall trend of the other compositions, as do the phase transition temperatures obtained from the changes in the gap-vs-temperature curves used to construct the phase diagram shown below.

**Figure 1:**
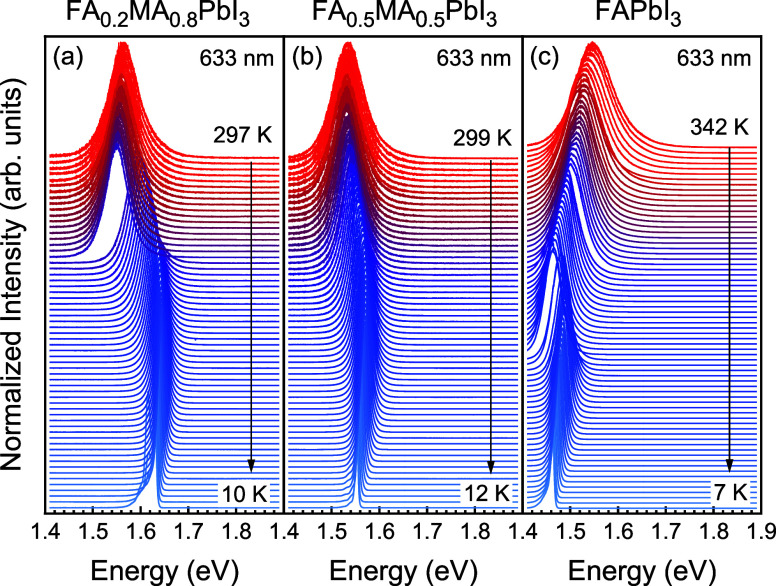
PL spectra of FA_*x*_MA_1–*x*_PbI_3_ single crystals for three representative compositions, namely, (a) *x* = 0.2, (b) *x* = 0.5, and (c) *x* = 1.0, recorded at different temperatures using the red line (633 nm) for excitation. The spectra were normalized to their maximum intensities and plotted with a vertical shift for increasing temperature. The temperature range is indicated (a temperature step of ca. 5 K).

**Figure 2:**
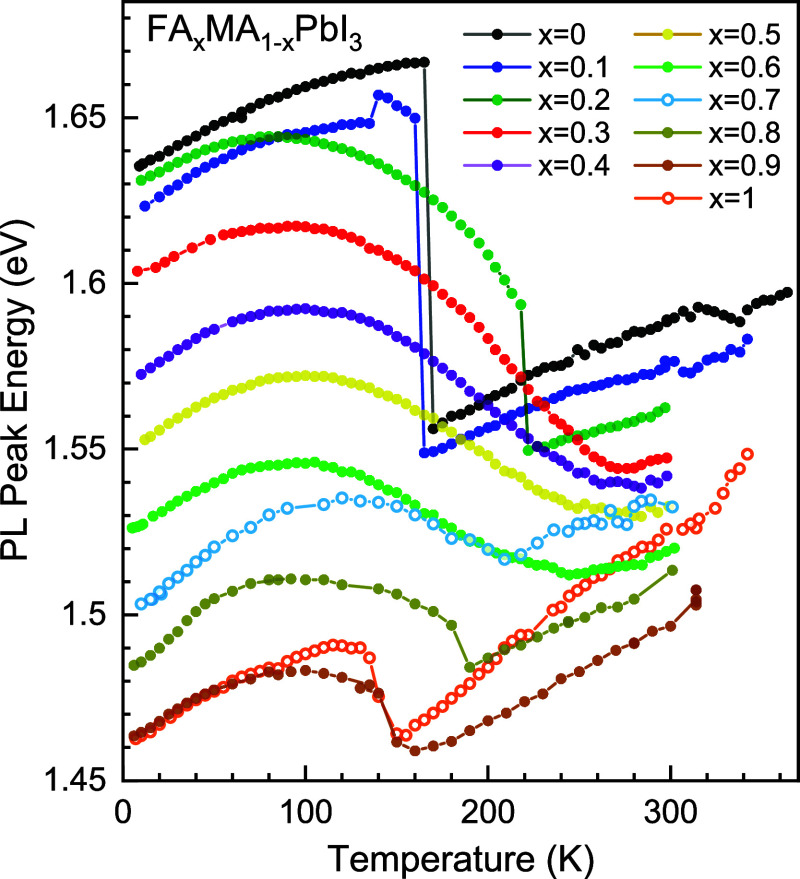
Maximum PL peak energy position plotted as a function of temperature, obtained from the PL line shape fits using a cross-product function (eq 1 of the Supporting Information) for the complete set of compositions of the FA_*x*_MA_1–*x*_PbI_3_ series.

Here we aim to elucidate the pronounced temperature-induced gap bowing in the low-temperature phases of FA_*x*_MA_1–*x*_PbI_3_ solid solutions, as displayed in Figure 2 and also observed elsewhere.^24^ We realized in a previous study on MAPbI_3_ ^14^ that it is more convenient for subsequent analysis to employ the first derivative of the gap with respect to temperature. For this purpose we derived numerically, point-by-point, the previously smoothed gap-vs-temperature curves of Figure 2. The results for all compositions are shown in Figure S0a–k on Note #3 of the Supporting Information. It is worth noticing, first, that in the low-temperature phases, the first derivatives are all pretty linear over a large temperature range of more than 100 K, indicating that the bowing is an almost perfect parabola. Second, the only first derivatives that do change sign, becoming negative in some temperature interval, are the ones for FA contents 0.2 ≤ *x* ≤ 0.9. Thus, it seems that a certain amount of disorder due to A-site cation mixing is necessary for having a sign turnover in the gap temperature dependence. To understand such a behavior we have first to disentangle the effects of thermal expansion and electron–phonon interaction on the gap temperature renormalization.^8,14,47^ According to eq 2 in Note #4 of the Supporting Information, the derivative of the gap over temperature contains solely the thermal expansion (TE) term and the one due to electron–phonon interaction (EP).^3,4,9^ The effect on the gap due to the lattice contraction with decreasing temperature is intimately related to the response of the electronic states under hydrostatic pressure:^3,4^

$${\left[\frac{\partial E_{g}}{\partial T}\right]}_{TE}=-\alpha_{V}\cdot B_{0}\cdot \frac{dE_{g}}{dP}$$ 1

where α_*V*_ is the volumetric thermal expansion coefficient, *B*_0_ is the bulk modulus and $\frac{dE_{g}}{dP}$ is the pressure coefficient of the gap, determined here from high-pressure experiments.

From the experimental point of view, the main achievement of this work is the determination of the gap pressure coefficient from PL spectra recorded at different pressures for a set of temperatures different (lower) than ambient. Since these experiments are very challenging and time-consuming, we were able to perform a restricted number of measurements at a few but selected temperatures in the range of the bowing for seven representative FA compositions. The main difficulty lies in the fact that the measurements must be performed within a very small pressure range up to 0.5–0.7 GPa, which, based on experience, is the range in which the first pressure-induced phase transition occurs,^48,49^ and this must be avoided. For subsequent measurements at a different temperature, the DAC often opens unintentionally, releasing the pressure medium and requiring a new helium (cryogenic) reload. The full set of results regarding the gap values versus pressure, as extracted from the PL spectra, are displayed for the selected temperatures in Figures S1–S7 in Note #4 of the Supporting Information for *x* = 0, 0.2, 0.4, 0.5, 0.6, 0.8, and 0.9, respectively. The slopes of the linear fits to the data points (red solid lines) directly give the gap pressure coefficients, which are listed in the corresponding Supporting Information Tables S1–S7. In good quantitative and qualitative agreement with previous observations for MAPbI_3_ ^18^ and MAPbBr_3_,^19^ for all studied FA compositions, the sign and magnitude of the gap pressure coefficient $\frac{dE_{g}}{dP}$ are strongly dependent on temperature, as is the TE term as well.

To calculate the TE term according to eq 1, we need the values of the volumetric thermal expansion coefficient α_*V*_ as a function of temperature, i.e., for the different structural phases adopted by the material and for all studied compositions. Unfortunately, this kind of data are only available for MAPbI_3_,^50–52^ FA_0.5_MA_0.5_PbI_3_ ^28^ and FAPbI_3_.^53^ Regarding the bulk modulus *B*_0_, the available information is similarly scarce; in fact, it is solely available for MAPbI_3_ ^54,55^ and FAPbI_3_ ^56^ and only at room temperature. However, the evidence is that *B*_0_ depends principally on the crystal structure, being temperature independent within the stability range of each phase.^54,56^ In short, we have been forced to make certain assumptions to compensate for the lack of information regarding α_*V*_ and *B*_0_, as explained in details in Note #4 of the Supporting Information. In Tables S1–S7 we have listed all the information needed to compute the TE term for the different compositions, temperatures, and crystal phases measured. Strikingly, strong variations in magnitude and even a change in sign of the TE term is observed at intermediate FA concentrations for the low-temperature (Ortho & Tetra-III) phases. However, we show below that these changes in the TE term only partially explain the temperature-induced bowing; the main contribution comes from changes in the electron–phonon interaction.

Regarding the contributions to the gap temperature renormalization stemming from electron–phonon interactions, the most important ones arise from peaks in the phonon density of states (DOS).^4^ This is at the origin of the Einstein-oscillator model,^9–11^ which approximates the different contributions to the EP term by oscillators with effective amplitude *A*_*i*_ and phonon eigen-frequency ω_*i*_, the latter inferred from the peaks in the phonon DOS (see eq 6 in Note #5 of the Supporting Information). The EP correction to the gap is obtained by calculating analytically the first derivative over temperature of the Bose–Einstein occupation factor *n*_*B*_(ω_*i*_, *T*) = (e^βℏω_*i*_^ – 1)^−1^, with β = $\frac{1}{k_{B}T}$ :

$${\left[\frac{\partial E_{g}}{\partial T}\right]}_{EP}=\underset{i}{\sum }\frac{A_{i}}{4T}\cdot \frac{\hbar \omega_{i}}{k_{B}T}\cdot \frac{1}{\sin\;h^{2}\left(\frac{\hbar \omega_{i}}{2k_{B}T}\right)}$$ 2

To evaluate the EP term, we thus have to perform a least-squares fit to the previously computed first-derivative data points using the sum of the TE and EP terms calculated according to eqs 1 and 2, respectively. The black-gray symbols in Figure 3a–c correspond to the first derivative data sets for a FA content of *x* = 0, 0.2 and 0.8; three representative compositions regarding the temperature-induced gap bowing (the results for the seven measured compositions are shown in Figures S8–S14 in Note #5 of the Supporting Information). To obtain the EP term (or terms) for each composition by fitting, the analysis of the first derivatives was performed piecewise within the temperature range of stability of the different phases, as indicated in the figures. The blue symbols and the blue dot-dashed curves in Figure 3a–c correspond to the TE term resulting from eq 1 and their polynomial fits, respectively, using the parameters tabulated in Tables S1–S7 on Note #4 of the Supporting Information. At high temperatures, i.e., for the Cubic and Tetra-I&II phases, the TE term is temperature-independent, as is common in MHPs.^13,17^ The analysis of the gap temperature dependence for these phases can be found in Note 5 of the Supporting Information. In the following, we will restrict the discussion exclusively to the behavior of the gap in the low-temperature phases, that is, the ortho and tetra-III phases, for which the bowing is usually observed.

**Figure 3:**
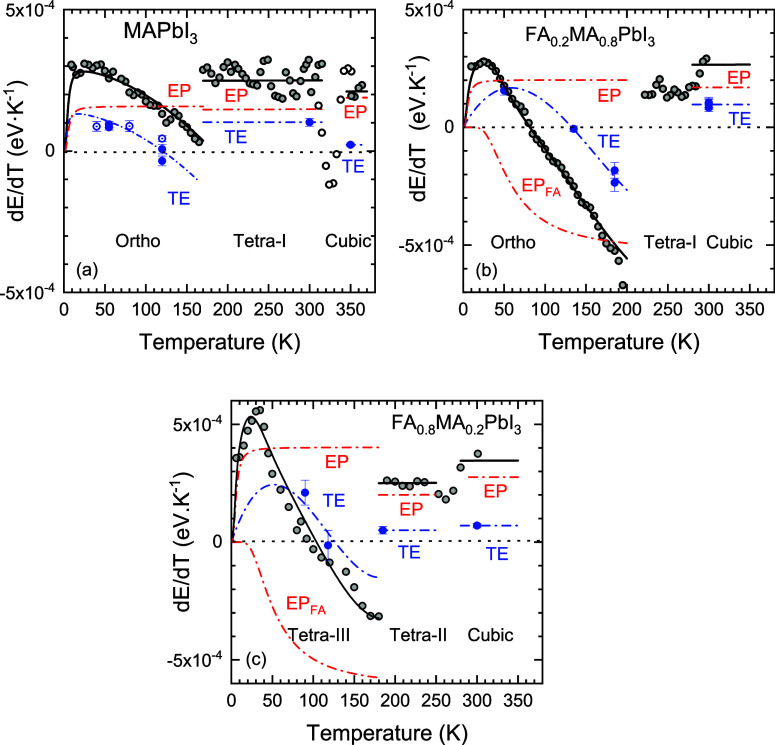
First derivative of the gap energy with respect to temperature (black-gray symbols), numerically calculated from the smoothed data of Figure 2 for FA_*x*_MA_1–*x*_PbI_3_ mixed-cation crystals with (a) *x* = 0, (b) *x* = 0.2 and (c) *x* = 0.8, three compositions representing different EP coupling cases. The solid black lines represent a fit to *only* the closed data points in the temperature range of stability of different phases, using the sum of thermal expansion (TE, blue dot-dashed curve and symbol) and electron–phonon interaction (EP, red dot-dashed curve). In (a), open blue symbols correspond to the values of the TE term calculated using the gap pressure coefficients determined for MAPbI_3_ at low temperatures.^18^ In (b) and (c), the curve labeled EP_FA_ represents the additional Einstein oscillator introduced to account for the anomalous FA-rattler related EP coupling. See text for details.

An inspection of the gap first-derivative curves shown in Figure S0a–k of the Supporting Information indicates that a turnover in the sign of the first derivative happens only for intermediate FA concentrations but not at the compositional ends (*x* = 0, 0.1, and 1). Let us thus start discussing MAPbI_3_ as representative of the latter case; the corresponding data are shown in Figure 3a. The contribution from electron–phonon interaction is calculated using the function of eq 2 and, like for the room-temperature Tetra-I phase,^14^ by considering a single Einstein oscillator with adjustable amplitude and frequency listed in Table 1. The solid black curve in Figure 3a represents the result of a least-squares fit to the data points of the ortho phase of MAPbI_3_ using the polynomial function describing the TE term (blue dotted-dashed curve) plus the single Einstein-oscillator function (dotted-dashed red curve).This oscillator also has a positive amplitude but a smaller frequency of 1.5 meV compared to that of the room-temperature oscillator (6 meV), as dictated by the lower occupation of higher energy phonons at low temperatures. This frequency coincides fairly well with the first peak in the phonon DOS of MAPbI_3_,^39^ corresponding to zone-edge acoustical phonon modes of the inorganic cage. It thus represents the *normal* EP coupling term.^13,14^ Interestingly, the nonlinearity in the gap first derivative of the ortho phase is entirely given by the temperature-dependent TE term (see Figure 3a).

**Table 1:** Einstein-Oscillator Parameters Corresponding to the Effective Amplitude *A*_*i*_ and Frequency *ω*_*i*_ Describing the Contribution from Electron–Phonon Interaction ${\left[\frac{\partial E_{g}}{\partial T}\right]}_{EP}$ to the Renormalization of the Band Gap for the Indicated Crystal Phase of FA_*x*_MA_1–*x*_PbI_3_ Single Crystals with the Seven FA Concentrations *x* Measured^a^

*x*	Phase	Einstein oscillator	*A*_*i*_ (meV)	ℏω_*i*_ (meV)	*T* fitting range (K)
0	Ortho	normal	2.7(5)	1.5(10)	<165
0.2	Ortho	normal	3.5(5)	1.5	<170
		FA rattler	–98(10)	16(1)	
0.4	Tetra-III	normal	6(1)	1.5	<130
		FA rattler	–85(10)	14(3)	
0.5	Tetra-III	normal	7(1)	1.5	<120
		FA rattler	–135(10)	18(2)	
0.6	Tetra-III	normal	6(1)	1.5	<125
		FA rattler	–125(10)	18(3)	
0.8	Tetra-III or Ortho	normal	7(1)	1.5	<125
		FA rattler	–105(10)	14(2)	
0.9	Tetra-III or Ortho	normal	5(1)	1.5	<110
		FA rattler	–75(10)	15(2)	

a The numbers in parentheses represent the error bars (uncertainty of the last digits) of the adjustable fitting parameters; conversely, numbers without error bars mean that this parameter was kept fixed during fitting. Also indicated is the temperature range in which the fit is excellent.

For intermediate FA concentrations the situation with the EP term changes dramatically. As can be seen in Figure 3b,c for *x* = 0.2 and 0.8, respectively (see Figures S10–S12 and S14 of the Supporting Information for the rest of compositions), at temperatures around 100 K, the gap first derivative strikingly changes sign from positive to negative. Although within the stability range of the Ortho and Tetra-III phases the contribution from the TE term can become negative above 100 K, its contribution is clearly insufficient to account for the strongly negative values of the (total) temperature gap derivative. Hence, the only way to correctly describe the gap temperature dependence is to introduce an additional Einstein oscillator with negative amplitude. For the fits, we thus used for the EP term two Einstein oscillators: One with positive amplitude and a fixed frequency of 1.5 meV, as for MAPbI_3_, to account for the normal EP coupling, and one with adjustable (negative) amplitude and frequency, called EP_FA_, for it will be assigned to FA rattler modes, as previously done for Cs in CsPbCl_3_ NCs.^22^ The red dot-dashed curves labeled EP_FA_ in Figure 3b,c and Figures S8–S14 of the Supporting Information represent the contribution of the additional Einstein oscillator. The results of the least-squares fits for the seven compositions studied but concerning only the EP terms are shown in Table 1 and plotted as a function of FA composition in Figure S15a,b of the Supporting Information. We note that the gap first derivatives are perfectly described by the fitting function (black solid curves) within the temperature range indicated in Table 1. Remarkably, the average frequency of the additional oscillator is (16 ± 2) meV, in excellent agreement with the lowest frequency FA libration (135 cm^–1^ = 17 meV) reported for FAPbI_3_ ^32^ or the average frequency of a mode with predominantly FA contribution and a fairly flat dispersion, indicating its localized nature, calculated for FAPbBr_3_.^17^ The necessary admixture of this FA-mode with the inorganic cage phonons can be observed in the piecewise finite dispersion and by the color variation of the dispersion curves shown in Figure 14c of ref 17, indicating a certain contribution from cage atoms, as expected for FA rattlers. The average amplitude of the additional Einstein oscillators is (−105 ± 25) meV, thus being the leading term that overcompensates the effects of thermal expansion and the normal EP term. All these facts and the clear similarity with the phenomenology exhibited by the Cs rattlers in Cs_*x*_MA_1–*x*_PbI_3_ single crystals (*x* = 0.05 and 0.1)^47^ and CsPb­(Br_1–*x*_Cl_*x*_)­Cl_3_ NCs with *x* > 0.5 ^22^ led us to the conclusion that the additional Einstein oscillator corresponds to FA-rattler modes which lead to the anomalous electron–phonon coupling that is the main cause of the observed gap bowing. We point out that, although direct spectroscopic evidence is lacking, rattling modes are considered the main source of the strong anharmonic phonon scattering leading to the ultralow thermal conductivity of MHPs.^57^

Recently, band-structure calculations of the temperature dependence of band gaps in disordered solids have advanced considerably thanks to the development of a nonperturbative approach based on the idea of searching for the polymorphic structure that minimizes total energy, taking into account the thermal occupation of the lattice phonons.^15–17,58^ In MHPs, polymorphism arises from dynamic disorder induced by the A-site cation dynamics.^59^ In brief, positional polymorphism represents multiple domains of local disorder at static-equilibrium that define a minimum in the system anharmonic potential energy surface (PES). Despite the presence of positional polymorphism, on a macroscopic level, the overall structure retains the average high symmetry of the tetragonal and cubic phases. For MHPs the simple monomorphous network typically utilized by ab initio electronic structure calculations is dynamically unstable, thus corresponding to a *local* minimum on the PES. For the exploration of the polymorphous structures a unified treatment was developed,^15^ which relies on the anharmonic special displacement method.^60^ This is an efficient supercell approach, where atoms are displaced according to a special linear combination of the computed phonon eigenvectors with amplitudes determined by the associated root mean squared displacements. It allows for the generation of a single optimal structure that best represents the system at thermal equilibrium. For the (high-temperature) tetragonal and cubic phases of a variety of 12 Pb and Sn halide perovskite compounds, it is found by averaging over ten polymorphous structures that the gap increases nearly linearly with temperature with an average slope of 4× 10^–4^ eV/K,^16,17^ often in excellent agreement with the experiment for materials featuring *normal* EP coupling (see literature survey in ref 13 and references therein). The question that immediately arises is how can a “static” band structure calculation account so well for an intrinsically “dynamic” property like the electron–phonon interaction and the temperature renormalization of the gap? The answer is simply found in the Born–Oppenheimer approximation which states that the lattice atoms are static for the crystal electrons, which move at speeds 3–4 orders of magnitude faster, just due to the extreme mass differences. Phonons just cause a local distortion of the lattice, which for crystal electrons does not vary in time but spatially. Statistically, the variations of the distortion of a given unit cell in time can be isomorphically mapped into the distortions of all other unit cells at a given time (snapshots of the phonon-induced deformations). Electrons just sample this polymorphous, spatially inhomogeneous structure, which is exactly the outcome of the polymorphous method. Regrettably, in the case of CsPbCl_3_, for which the gap temperature slope at ambient was shown to be dominated by the effects of anomalous EP coupling,^22^ the polymorphous approach delivers a small though still positive slope.^17^ This might be an indication that rattler modes are not being effectively incorporated into the polymorphous methodology.

What is also well explained within the polymorphic method is the significant reduction of the band gap as MA is gradually replaced by FA (see Figure 2); its explanation remained elusive until now. Although the molecular orbitals of the organic cations play no role in defining the band-edge states, the A-site cation size and polarity strongly affect the amount of local disorder (particularly octahedral tilting) present in the polymorphous phases. The larger the cation size, the less disorder is locally generated, which leads to a smaller (positive) gap renormalization. The fact that the gap strongly opens with octahedral tilting is, for example, directly inferred from the observed jumps in the gap at the phase transition from Tetra-I/Tetra-II to Ortho (see Figure 2 and ref 61). As compared with the monomorphous crystal structure calculated by density-functional theory (DFT), the gap opening due to local disorder computed within the polymorphous model is 0.27, 0.46, and 0.61 eV in FA-, MA-, and Cs-based compounds, respectively.^17^ This implies a gap difference between MAPbI_3_ and FAPbI_3_ of 190 meV, in very good agreement with experiment (see Figure S15c on Note #5 of the Supporting Information). To be fair, a similar composition dependence of the gap has been previously reported for the FA_*x*_MA_1–*x*_PbI_3_ system, as computed by first-principles DFT calculations for structures where the orientations of the organic cations resulted from careful examination of the energy landscape.^62^ These (zero-temperature) calculations indicate that H bonding is crucial for the stabilization of those crystal structures, which exhibit an increment in Pb–I–Pb bond angle from ca. 160° to 175°, as FA content varies from 0 to 1.^62^ Concomitantly with this decrease in average octahedral tilting, which leads to a more symmetric structure, the gap decreases linearly with increasing FA content, being the gap difference between the two end compositions ca. 300 meV, when spin–orbit coupling is considered.

To better understand the conditions leading to anomalous EP coupling, we will now revisit the composition–temperature phase diagram of FA_*x*_MA_1–*x*_PbI_3_ in light of new theoretical developments. This phase diagram was recently mapped out using a machine-learned interatomic potential in molecular dynamics (MD) simulations.^26^ The main result was the identification of a morphotropic phase boundary (labeled MPB1 in Figure 4) at around 27% FA content, which separates the two tetragonal phases, named Tetra-I and Tetra-II, characteristic of both compositional end materials: MAPbI_3_ and FAPbI_3_, respectively. A key observation is that the Tetra-I and Tetra-II phases exhibit opposing tilt patterns (in Glazer notation, out-of-phase *a* ^0^ *a* ^0^ *c* ^–^ vs in-phase *a* ^0^ *a* ^0^ *c* ^+^); patterns which coincide with the Brillouin-zone edge R_*z*_ and M_*z*_ phonon mode eigenvectors (*z* is the long tetragonal axis), respectively. The transition from one phase to another is not abrupt but gradual and coincides with a mode crossover at which R and M phonons become nearly degenerate.^26^ An analysis of the evolution of the phonon mode projections indicate that, across the transition, there is a certain range of compositions around the MPB where the material breaks up into a mosaic-like arrangement of layered regions in which either R_*z*_ or M_*z*_ modes are predominantly activated. Interestingly, from the point of view of the tilt patterns, the FA_*x*_MA_1–*x*_PbI_3_ solid solutions behave as an eutectic system.^63^ Below the solidification point, a eutectic exhibits a symmetric phase diagram with two MPBs at compositions *x*_0_ and 1 – *x*_0_, separating the two solid phases of the pure materials from the intermediate region of coexistence of both phases. As depicted in the revised phase diagram of Figure 4, we thus suggest that the intermediate phase is the Tetra-III = Tetra-I + Tetra-II, with MPBs at approximately 27% and 73% FA content, obtained from the onset temperatures of the gap bowing. For completeness and according to recent experimental and theoretical work in FA_*x*_MA_1–*x*_PbBr_3_ ^64^ and FAPbI_3_,^31^ respectively, we also speculate that the low-T phase of FA-rich compounds may be the Ortho phase with *Pnma* symmetry of MA-rich compounds. We note that the transition from Tetra-II to (now) Ortho phase for *x* ≥ 0.7 is characterized by a discontinuity in the gap energy as for MA-rich compounds. In contrast, the Tetra-III/Ortho phase boundary is marked with dashed line in Figure 4 because its actual position is just conjectural. Regrettably, Raman data were inconclusive in this respect due to a strong PL signal, arising by excitation with the 785 nm laser (1.58 eV) that was above the gap for *x* > 0.4 (see the Supporting Information of ref 25).

**Figure 4:**
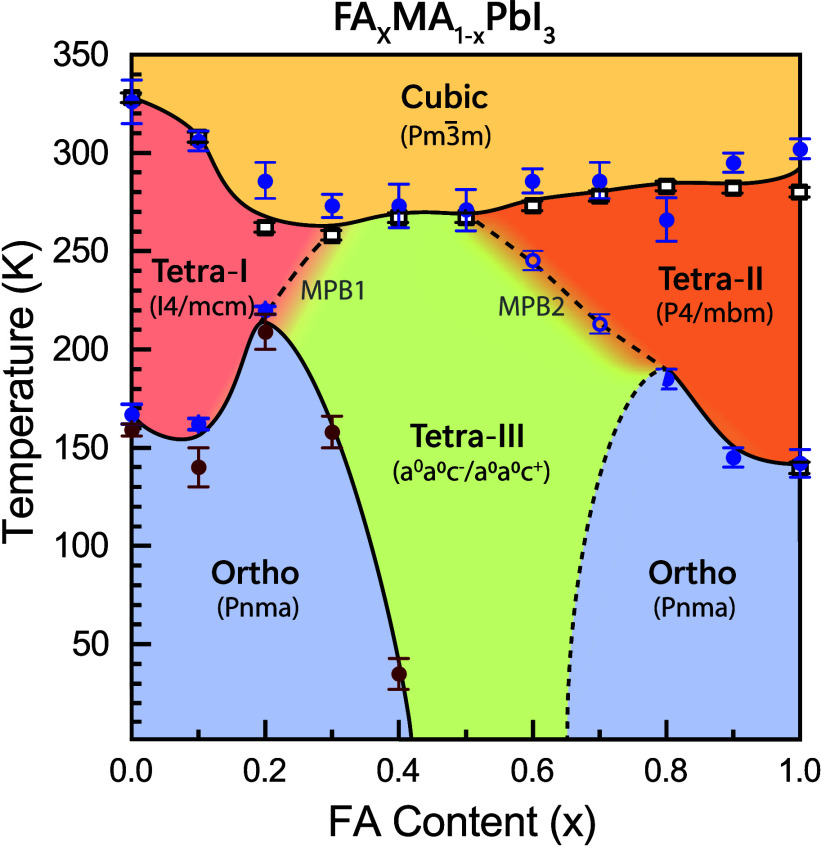
Revised version of the temperature-versus-composition phase diagram of FA_*x*_MA_1–*x*_PbI_3_ perovskite solid solutions.^25^ The symbols represent experimental data (Raman and PL data from ref 25 and X-ray/neutron scattering data from refs 28, 29, 36, and 37). The different crystal structures and their symmetry are indicated. Phase separation lines as well as morphotropic phase boundaries (MPBs), indicated by dashed lines, are a guide to the eye.

A highly relevant result from MD simulations performed for a very large supercell is the formation of twin domains with alternating preferential tilt axis parallel to the long tetragonal axis, with 90° domain walls along the ⟨011⟩ direction.^26^ The aforementioned domains form as a mechanism for strain relaxation and are actually phenomenologically the same as the ferroelastic twin domains appearing in the low-temperature orthorhombic phase upon transition from the tetragonal phase (see, for example, videos of this transformation in MAPbBr_3_ single crystals in the Supporting Information of ref 65). The presence of ferroelastic domains seems to be key for the activation of the anomalous EP term associated with the FA-rattler modes. This is so because the additional Einstein oscillator of the FA rattlers and the large gap bowing are signatures solely of the Tetra-III and Ortho phases, whereas the homogeneous Cubic and Tetra-I,II phases exhibit a linear gap temperature dependence characteristic of a normal EP coupling mechanism.^13,17^ For an in-depth discussion of the tight correlation between ferroelastic-domain formation and the immediate appearance of a negative-amplitude Einstein oscillator in the EP term, we refer to Note #6 of the Supporting Information. Furthermore, from DFT calculations performed for representative snapshots of MD simulations at 330 K, the standard deviation of the valence and conduction band edges was computed as a function of FA composition.^26^ The magnitude of the fluctuations is a measure of the strength of the electron–phonon interaction, as experimentally demonstrated by gap fluctuations upon THz or infrared phonon excitation in MAPbI_3_ ^66,67^ and BA_2_PbI_4_.^67^ The fluctuations are maximal around the MPB, indicating an enhancement of the EP coupling, compatible with the activation of the anomalous EP term, in the phases with twin domains.

In conclusion, by incorporating recent experimental^64^ and theoretical^26^ results, we have revisited the phase diagram of the FA_*x*_MA_1–*x*_PbI_3_ system, linking structure, lattice and cation dynamics as well as the electronic band structure, with special emphasis on the understanding of the dependence on temperature of the band gap. In particular, we have elucidated the reason for the pronounced bowing in the gap temperature dependence observed in the low-temperature phases (below ca. 250 K) of the solid solutions for intermediate FA compositions 0.2 ≤ *x* ≤ 0.9. For this purpose we carried out measurements of the gap pressure coefficient at cryogenic temperatures, using He as the pressure transmitting medium, to univocally disentangle the contributions stemming from thermal expansion and electron–phonon interaction. In the Tetra-III and Ortho phases, where the bowing is observed, despite the contribution of the strongly temperature-dependent TE term, an accurate description of the gap bowing is only obtained by invoking the activation of an anomalous EP term represented by an Einstein oscillator with average frequency and coupling strength of 16 ± 2 and −105 ± 25 meV, respectively. In analogy to Cs rattlers,^22,47^ the modes leading to the anomalous coupling are identified as FA rattlers, which are a combination of inorganic-cage phonons involving octahedral tilting in synchrony with low-frequency FA librations.^17,32^ Although the microscopic mechanism is unknown, the formation of ferroelastic stripe domains with 90° alternating orientation of the preferential tilt axis (coinciding with the long tetragonal or orthorhombic crystal axis) appears to be crucial for the activation of the FA rattlers. These domains naturally form in mixed-cation solid solutions after the transition into the Tetra-III or Ortho phases as a means to minimize the macroscopic strain in the sample. Given the relevance of the gap temperature dependence for the optoelectronic properties of semiconductors in general, and perovskites in particular, its correct assessment is key for the advancement of emergent photovoltaic, efficient light emission, and/or sensing devices, for instance.

## Supplementary Material



## Data Availability

All data generated or analyzed during this study either are included in this published article and its Supporting Information or are available from the corresponding author on reasonable request.

## Notes

Details about the synthesis of the mixed-cation perovskites and the PL measurements as a function of temperature and pressure and of both simultaneously; first-derivative plots, as obtained by point-by-point derivation of the gap-vs-temperature curves; detailed analysis of the literature data and the measured gap pressure coefficients so as to determine the TE term; complete set of the least-squares fits to determine the EP terms; discussion of the correlation between ferroelastic-domain formation and anomalous EP coupling (PDF)
