# Supplementary material for: Origin of the Temperature-Induced Gap Bowing of Formamidinium–Methylammonium Lead Iodide Perovskites: Role of Cationic Rattlers
Source: J Phys Chem Lett. 2026 Jul 28;17(31):9097–106. doi: 10.1021/acs.jpclett.6c01965 (PMC13455107; doi:10.1021/acs.jpclett.6c01965)
Supplement: Supplementary file 1 [file jz6c01965_si_001.pdf]

Supporting Information

Origin of the Temperature-Induced Gap Bowing of Formamidinium-Methylammonium Lead Iodide Perovskites: Role of Cationic Rattlers

Kai Xu,[†] Adrián Francisco-López,[†] Bethan L. Charles,[‡,¶] M. Isabel Alonso,[†] Miquel Garriga,[†] Mark T. Weller,[‡,§] and Alejandro R. Goñi[*,†,||]

†*Institut de Ciència de Materials de Barcelona (ICMAB-CSIC), Carrer dels Til.lers, 08193 Cerdanyola del Vallès, Spain*

‡*Dept. of Chemistry & Centre for Sustainable Chemical Technologies, University of Bath, Claverton Down, Bath BA2 7AY, UK*

¶*School of Electrical, Electronic and Mechanical Engineering, Queens Building, University of Bristol, Bristol BS8 1TR, UK*

§*Dept. of Chemistry, Cardiff University, Wales CF10 3AT, UK*

||*ICREA, Passeig Lluís Companys 23, 08010 Barcelona, Spain*

E-mail: goni@icmab.es

Note ♯0: $FA_xMA_{1-x}PbI_3$ crystal growth

The inverse solubility method of Saidaminov *et al.*[1] was developed to produce crystals of $FA_xMA_{1-x}PbI_3$ across the full composition range. Stoichiometric quantities of MAI, FAI (Great-

Cell Solar) and PbI_2 (Merck, 99%) were dissolved at 60 °C in dry γ-Butyrolacetone (Alfa Aesar). Concentrations of the precursor solutions were kept at 1 M, with an exception for the FA rich materials $FA_xMA_{1-x}PbI_3$ ($x \geq 0.8$), where concentrations were lowered to 0.9 M to allow the FAI to fully dissolve. When the precursor salts and lead halide powders had completely dissolved, the solutions were quickly filtered using Millex GS filters of pore size 0.22 μm and transferred to clean cylindrical 4 ml glass vials. The solutions were then heated to 110 °C at a rate of 10 °C hour^{-1} using an oil bath, the temperature of which was carefully controlled and monitored using a Stuart SCT1 controller. Solutions were maintained at 110 °C for 2 hours allowing crystallization, the temperature was then raised to 120 °C for a final hour before the remaining solution was filtered and large single crystals were oven dried at 100 °C overnight.

Note ♯1: Temperature and pressure-dependent photoluminescence (PL) experiments

The PL spectra were excited with the 633 nm line of a He-Ne laser using a very low incident light power below 2 μW (power density <15 W/cm^2) to avoid any photo-degradation of the samples.[2] Spectra were collected using a 20× long working distance objective with NA=0.35 (laser spot of ca. 4 μm in diameter) and dispersed with a high-resolution LabRam HR800 grating spectrometer equipped with a charge-coupled device detector. PL spectra were corrected for the spectral response of the spectrometer by normalizing each spectrum using the detector and the 600-grooves/mm grating characteristics. Temperature-dependent PL measurements on large single crystals exhibiting flat surfaces were carried out by decreasing the temperature between ca. 365 and 6 K in steps of 5 K, using liquid helium in a gas flow cryostat from CryoVac with optical access that fits under the microscope of the Raman setup.

Room temperature high-pressure PL measurements in the range up to 1 GPa were performed employing a gasketed diamond anvil cell (DAC). Anhydrous propanol was used as pressure transmitting medium which ensures perfect hydrostatic conditions[3] up to 4.2 GPa and proved chem-

ically inert to (FA,MA)PbI_3. For loading the DAC, small chips with a thickness below ca. 30 μm were produced by crushing a big single crystal between two glass slides. By close inspection of the debris we were able to pick up small enough, good-quality single crystals, recognized by their flat and shiny surface under the microscope. Small pieces of less than 100×100 μm^2 in size were placed into the DAC together with a ruby sphere for pressure calibration.[4] Here we point out that the Inconel gasket was intentionally pre-indented to a fairly large thickness of 120 μm, before drilling a hole of ca. 250 μm with a spark-gap machine from EasyLab. The reason was to be able to adjust the pressure with the DAC in steps less than 0.05 GPa, mainly at very low pressures (below 1 GPa). For this purpose an electric motor drive was used to change the pressure in a continuous manner and at low speed (by ca. 0.05 GPa/min).

In contrast, for the high-pressure PL measurements at **low temperatures** down to 4 K a specially designed He-bath cryostat (Konti-IT from Cryovac) was used. The cryostat can allocate the DAC of Syassen/Holzapfel type in its cold bore while granting optical access.[5] Two steel rods designed to fit the threaded rods of the DAC inside the cryostat allow the pressure tuning without removing the DAC from the cryostat. When using the DAC at low temperatures with He as a pressure medium, the loading of the cell proceeds inside the cryostat. The cell is immersed in fluid He, which is pumped to lower its equilibrium temperature till the transition to the superfluid state at 2.17 K occurs. Under this condition, He easily enters into the cell, filling it completely and the DAC is remotely closed. As He instantly crystallizes when subjected to pressure at 2 K, the cell has to be heated well above the melting point to relax non-hydrostatic strain generated by closing the DAC. When cooling down again into the solid phase it is assumed that the pressure on the sample is mostly hydrostatic. To avoid the generation of unwanted uniaxial stress components on the sample, pressure is always changed, if possible, after heating the DAC to a temperature above the solid-liquid phase transition of He. Pressure is monitored in situ using the aforementioned ruby fluorescence method[4] with pertinent temperature correction.[6]

Note ♯2: Analysis of PL spectra

Figures S1(a-k) of the Supporting information of the work of Francisco-López et al.[7] show the complete set of PL spectra measured at different temperatures between ca. 4 and 385 K for all eleven compositions of $FA_xMA_{1-x}PbI_3$ mixed crystals, spanning the whole compositional range from $x = 0$ to 1 in steps of 10% FA content. In the main manuscript we only show the corresponding spectra cascades for x=0.2, 0.5 & 1. The main emission peak observed for all compositions at higher temperature was assigned to free-exciton (FE) recombination.[9–12] Thus, for the quantitative analysis of the PL spectra we performed a lineshape fitting[13] using a Gaussian-Lorentzian cross-product function (lineshape fitting examples were published elsewhere[8]). Its expression reads as:

$$G \times L(\hbar\omega) = A \cdot \frac{\Gamma^2}{4 \cdot s \cdot (E_0 - \hbar\omega)^2 + \Gamma^2} \cdot \exp\left(-4 \cdot \ln 2 \frac{(1-s) \cdot (E_0 - \hbar\omega)^2}{\Gamma^2}\right) \tag{1}$$

where A is the amplitude prefactor, E_0 is the peak energy position, Γ is the full width at half maximum (FWHM) and s is a weight parameter which takes the value $s = 0$ for pure Gaussian lineshape and $s = 1$ for pure Lorentzian. The values of these four adjustable parameters were obtained as a function of temperature from lineshape fits to the measured PL spectra using Eq. (1). For the cases where there is a coexistence of two phases we used two cross-product functions with independent variable parameter sets. The much more complicated analysis of the wealth of peaks, apparent at low temperatures (below ca. 70 K) for high MA content and ascribed to radiative recombination of bound (acceptor/donor) exciton complexes, was published elsewhere.[14] For all compositions the peak energy position E_0 which can be taken as representative of the band gap of the material, exhibits a clear and pronounced bowing at intermediate to low temperatures. In order to understand the origin of this bowing, in the following we will disentangle the contributions to the gap temperature dependence stemming from thermal expansion and electron-phonon interaction at selected temperatures and for some of the eleven compositions.

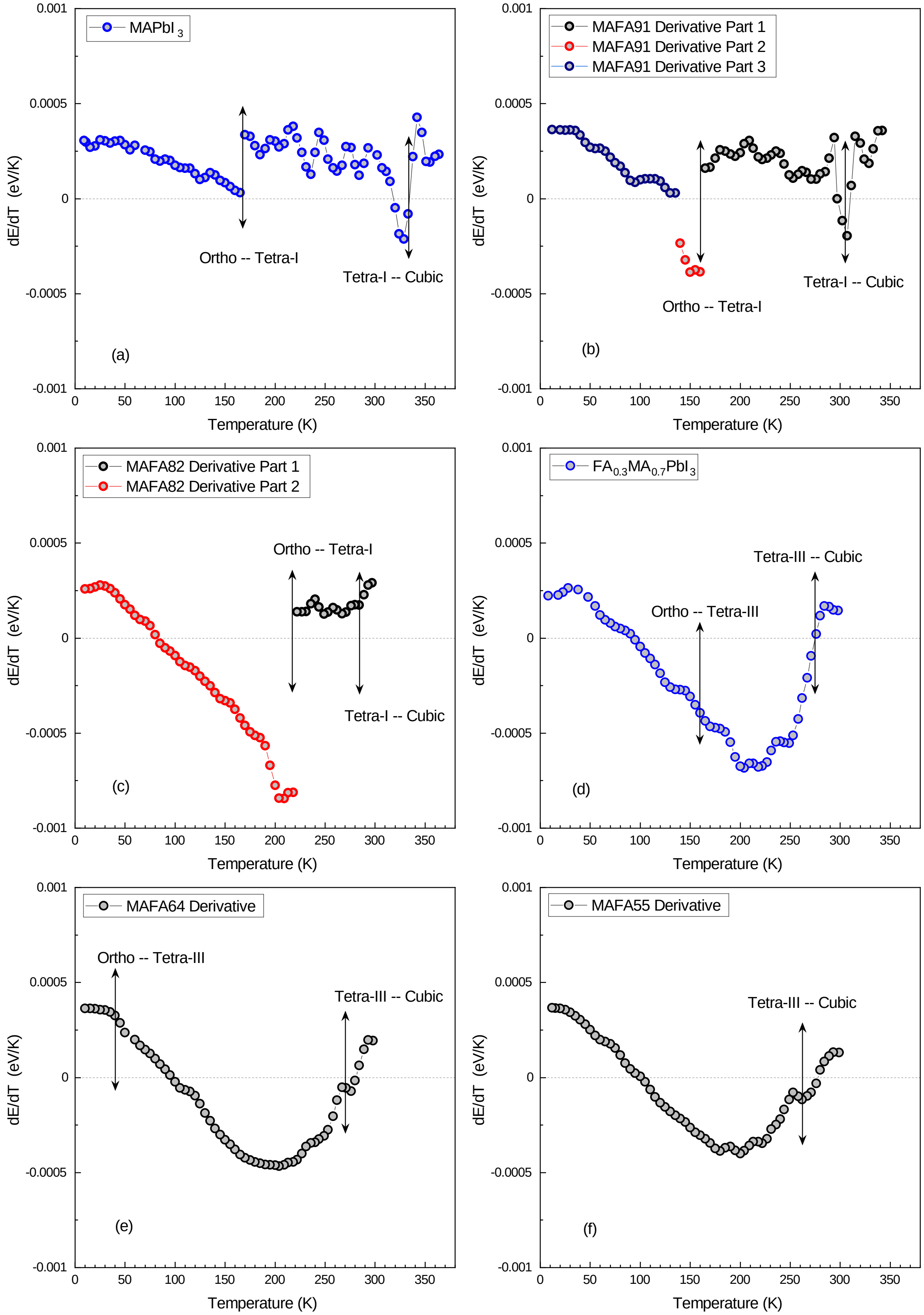
MAPbI 3
Ortho -- Tetra-I
Tetra-I -- Cubic
(a)
MAFA91 Derivative Part 1
MAFA91 Derivative Part 2
MAFA91 Derivative Part 3
Ortho -- Tetra-I
Tetra-I -- Cubic
(b)
MAFA82 Derivative Part 1
MAFA82 Derivative Part 2
Ortho -- Tetra-I
Tetra-I -- Cubic
(c)
FA0.3MA0.7PbI3
Ortho -- Tetra-III
Tetra-III -- Cubic
(d)
MAFA64 Derivative
Ortho -- Tetra-III
Tetra-III -- Cubic
(e)
MAFA55 Derivative
Tetra-III -- Cubic
(f)
dE/dT (eV/K)
Temperature (K)
0.001
0.0005
0
-0.0005
-0.001
0
50
100
150
200
250
300
350

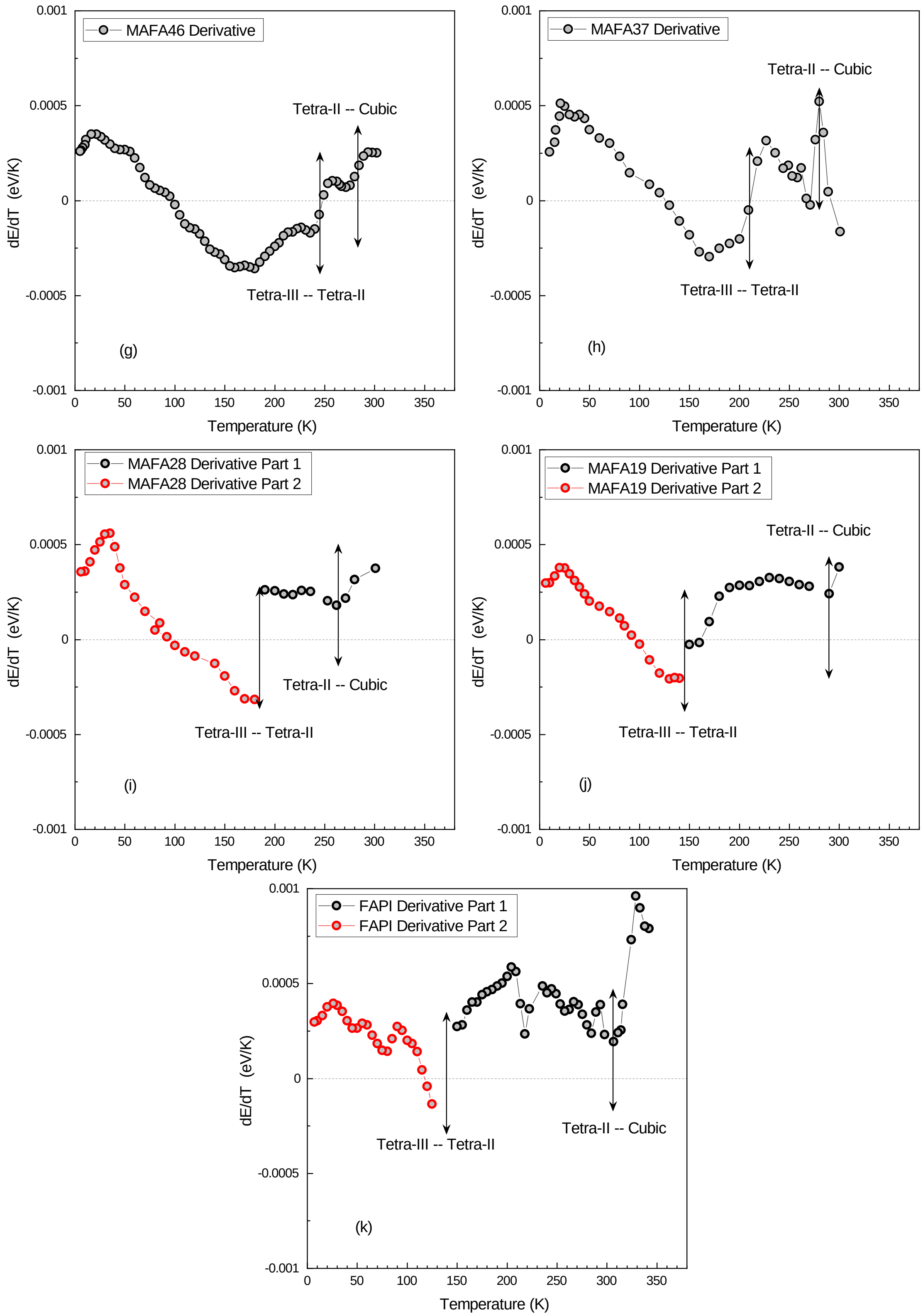

Figure S 0: First derivative of the PL peak energy E_0 with respect to temperature, plotted as a function of temperature, for the $FA_xMA_{1-x}PbI_3$ series with (a) $x = 0$, (b) 0.1, (c) 0.2, (d) 0.3, (e) 0.4, (f) 0.5, (g) 0.6, (h) 0.7, (i) 0.8, (j) 0.9, and (k) $x = 1$. The temperatures at which different structural phase transitions occur are indicated by arrows.

Note ♯3: Temperature dependence of the gap energy & its first derivative

The values of the fitting parameter corresponding to the peak energy E_0, which we take for the gap energy, as obtained from the line shape fits using Eq. (1), is plotted as a function of temperature in Fig. 2 of the main manuscript for the eleven compositions of $FA_xMA_{1-x}PbI_3$ studied. In Figs. S0(a)-(k) we illustrate the procedure to construct the graphs of the derivative of E_0 over temperature plotted as a function of temperature for each FA concentration $x \in [0,1]$ in steps of 0.1, respectively. In order to avoid the amplification of the scatter of the data points when taking the first derivative numerically and point-by-point, we have previously smoothed the E-vs-T curves (see procedure, for example, in the Supporting Information of Ref. [15]). These first-derivative plots will be very important for disentangling the effects of thermal expansion and electron-phonon coupling, as well as for the discussion of the structural behavior of the $FA_xMA_{1-x}PbI_3$ solid-solution series. The temperatures at which different structural phase transitions occur are indicated by arrows. For low FA concentrations the temperature of the phase transition between the cubic and the tetra-I phase (the same procedure is valid for the cubic-tetra-II transition for high FA contents), which in the E_0-vs.-T curve corresponds to a zigzag type behavior, is extracted from the zero of the first derivative curve, as indicated in Fig. S0(a), for example. In the same figure, we also show the determination of the transition temperature for the tetra-I to orthorhombic transformation, which simply corresponds to the temperature at which the jump in the first derivative occurs. For the smooth transition between the cubic and the tetra-III phase, taking place for intermediate FA concentrations, the transition temperature is obtained again from the crossing point of the first derivative curve and the temperature axis (see example in Fig. S0(c)). This corresponds to the inflection point in the E_0-vs.-T curve, which is less straightforward to be determined.

Note ♯4: The thermal expansion (TE) term

The derivative of the gap over temperature contains two terms; one accounts for thermal expansion effects (TE) and the other corresponds to the renormalization directly caused by electron-phonon interaction (EP), which includes the Debye-Waller and self-energy corrections:[16–18]

$$\frac{dE_g}{dT} = \left[\frac{\partial E_g}{\partial T}\right]_{TE} + \left[\frac{\partial E_g}{\partial T}\right]_{EP}. \tag{2}$$

The effect on the gap due to the contraction of the lattice with decreasing temperature is intimately related to the response of the electronic band structure upon application of external hydrostatic pressure. It thus holds[16,17,19]

$$\left[\frac{\partial E_g}{\partial T}\right]_{TE} = -\alpha_V \cdot B_0 \cdot \frac{dE_g}{dP}, \tag{3}$$

where α_V is the volumetric expansion coefficient, B_0 is the bulk modulus, i.e. the inverse of the compressibility, and $\frac{dE_g}{dP}$ is the pressure coefficient of the gap, which can be determined from high pressure experiments. In the entire temperature range of the experiments, α_V is positive and the sign of Eq. (3) for the thermal expansion contribution is determined by the sign of the pressure coefficient. For most metal halide perovskites (MHPs) direct gaps $\frac{dE_g}{dP}$ is negative around room temperature, hence, thermal expansion causes a gap increase.[20] The negative sign of $\frac{dE_g}{dP}$ arises from the inverted atomic orbital character of the states at the top and bottom of the valence and conduction band of lead halide perovskites, respectively, as explained elsewhere.[8] At lower temperatures than ambient the situation can fundamentally change, where positive gap pressure coefficients are measured, which lead to a reduction of the gap with temperature. In fact, this is the case of the Tetra-III phase of the $FA_xMA_{1-x}PbI_3$ mixed crystals at intermediate temperatures for which the pronounced gap bowing is observed.

For the determination of the TE term, according to Eq. (3), we first need to search the literature looking for values of the volumetric thermal expansion coefficient of the solid sotutions as a function of temperature. Fortunately, we found data for the two extreme compositions ($MAPbI_3$

& $FAPbI_3$) and the intermediate one ($FA_{0.5}MA_{0.5}PbI_3$). For the rest of the compositions we were forced to interpolate the α_V data making sensible assumptions, as indicated below. In Table S0 we summarize the thermal expansion coefficient values $\alpha_V(T)$ from the cited sources, indicating the temperature range for which each value is valid. We point out that the listed $\alpha_V(T)$ were obtained from the measured unit-cell volumes $V(T)$ by computing the slope $\frac{1}{V(T)} \cdot \frac{V(T+\Delta T)-V(T)}{\Delta T}$ stepwise for each temperature range indicated in Table S0.

Table S 0: Computed volumetric thermal expansion coefficient $\alpha_V(T)$ as a function of temperature for $MAPbI_3$, $FA_{0.5}MA_{0.5}PbI_3$ and $FAPbI_3$ obtained from the measured volume-vs-temperature data of the cited literature. Also indicated are the different temperature ranges of validity of α_V and the corresponding stable phase.

x	α_V (10^{-4} K^{-1})	Phase	Temperature range (K)	Reference
0	1.41	Cubic	325 - 375	21
	1.27	Tetra-I	160 - 320	22
	1.56	Ortho	140 - 155	23
	1.14	Ortho	85 - 140	23
	1.01	Ortho	55 - 85	23
	0.81	Ortho	30 - 55	23
	0.26	Ortho	10 - 30	23
0.5	1.54	Cubic	275 - 300	24
	2.30	Tetra-III	150 - 275	24
1	1.08	Cubic	300 - 500	25
	1.90	Tetra-II	150 - 300	25
	1.75	Tetra-III	90 - 150	25

We now turn to the discussion of the bulk modulus B_0 and its possible dependence on composition and temperature. With respect to the temperature dependence of B_0, the situation is very unsatisfactory, since, to the best of our knowledge, there are almost no data available, not even for the archetypal perovskite $MAPbI_3$. The only solution is to use, for each of the phases the material adopts as a function of temperature, the B_0 value obtained for the corresponding high-pressure phase at room temperature. This assumption is supported by temperature-dependent de-

terminations of the Young modulus of $FAPbI_3$,[26] which indicate that the elastic properties of that perovskite are essentially constant in the entire temperature range of stability of each crystalline phase. Furthermore, a study of the elastic properties of four MA metal halides, namely $MAPbI_3$, $MAPbBr_3$ and $MAPb(Br_xI_{1-x})_3$ with x=0.2 & 0.6 has shown that there is no clear systematics in composition for the bulk moduli of these compounds, being all within experimental uncertainty.[27] In conclusion, to compute the TE term we have used the same bulk modulus value for all cubic, all tetragonal and all orthorhombic phases, that is B_0 values independent of temperature and composition. The actual numbers correspond to the accurate values determined for $MAPbI_3$ at high pressures but room temperature:[28] 16(3) GPa for the tetragonal-I phase stable at ambient, 13(5) GPa for the cubic phase stable between 0.4 and 3 GPa and 30(2) GPa for the orthorhombic phase stable above 3 GPa. Numbers in parentheses are the error bars.

Finally, according to Eq. (3), the gap pressure coefficients at given temperatures, $\frac{dE_g}{dP}(T)$, are needed for the assessment of the TE term at that temperatures. For this purpose, we run high-pressure PL experiments at cryogenic temperatures using He as pressure-transmitting medium. Since these are extremely complex and time-consuming experiments, we performed the measurements for only seven of the eleven studied solid-solution compositions and for a small set of representative temperatures. The gap pressure coefficient of Eq. (3) is determined from the slope of the energy versus pressure curves, where the data points were previously obtained from fits to the PL spectra recorded at different pressures.

Figure S1 shows the variation with pressure of the gap energy of $MAPbI_3$, as obtained from PL line-shape fits of the PL spectra measured at low pressure and at the selected temperatures. The slopes of the linear fits to the data points (red solid lines in Fig. S1) are listed in Table S1 together with the values of B_0 and α_V employed to calculate the thermal expansion term TE in the case of $MAPbI_3$. We have also included the only available data, other than the ones reported here, concerning the pressure coefficient of the gap in the orthorhombic phase.[29]

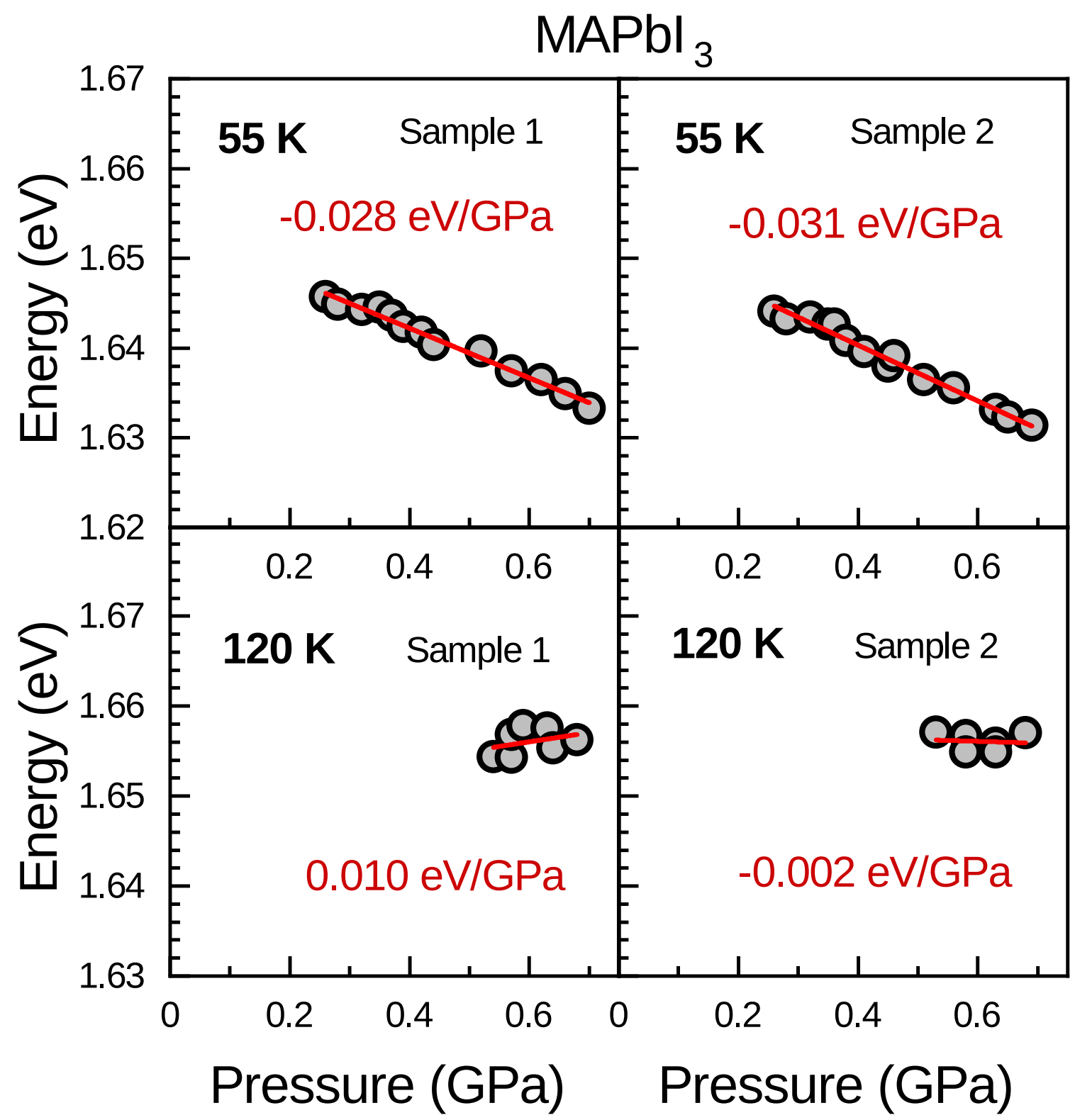

Figure S 1: The energy of the gap extracted from the PL peak maximum (symbols) plotted as a function of pressure and for different temperatures, as indicated, in the case of $MAPbI_3$. The red lines represent a fit to the data points using a linear function. The slope values are also indicated.

Table S 1: Computed thermal expansion term $\left[\frac{\partial E_g}{\partial T}\right]_{TE}$ from the values of the volumetric thermal expansion coefficient $\alpha_V(T)$, bulk modulus B_0 and gap pressure coefficient $\frac{dE_g}{dP}$ for $MAPbI_3$ and different temperatures T. Numbers in parentheses are the error bars. The asterisks indicate that the data is from Ref. [29] and the dagger indicates that B_0 is from the room-temperature high-pressure phase (Ref. [28]).

	**$MAPbI_3$**				
Phase	T (K)	α_V (10^{-4} K^{-1})	B_0 (GPa)	$\frac{dE_g}{dP}$ (eV/GPa)	$\left[\frac{\partial E_g}{\partial T}\right]_{TE}$ (10^{-4} eV/K)
Ortho	40*	0.81(5)	30(6)	-0.036(6)*	0.87(12)
	55	1.01(5)	30(6)	-0.028(3)	0.85(9)
	55	1.01(5)	30(6)	-0.031(3)	0.94(9)
	80*	1.01(5)	30(6)	-0.029(1)*	0.88(6)
	120	1.14(5)	30(6)	0.010(10)	-0.34(15)
	120	1.14(5)	30(6)	-0.002(10)	0.07(15)
	120*	1.14(5)	30(6)	-0.013(2)	0.44(5)
Tetra-I	300	1.27(5)	16(3)	-0.050(10)	1.02(4)
Cubic	350	1.41(5)	13(5)†	-0.012(10)	0.22(6)

Figure S2 shows the variation with pressure of the gap energy of $FA_{0.2}MA_{0.8}PbI_3$, as obtained from PL line-shape fits of the PL spectra measured at low pressure and at the selected temperatures. The slopes of the linear fits to the data points (red solid lines in Fig. S2) are listed in Table S2 together with the values of B_0 and α_V employed to calculate the thermal expansion term TE.

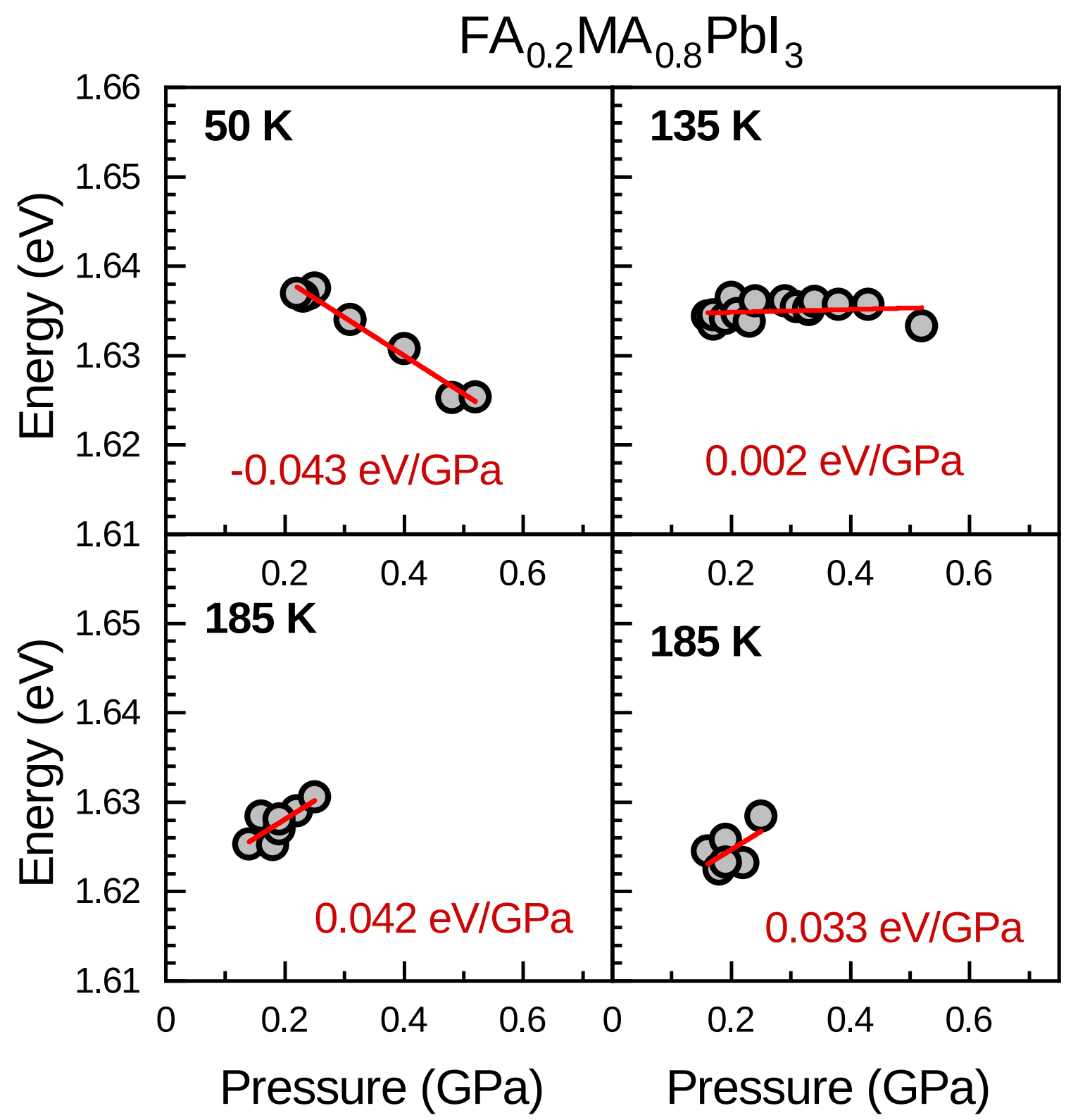

Figure S 2: The energy of the gap extracted from the PL peak maximum (symbols) plotted as a function of pressure and for different temperatures, as indicated, in the case of $FA_{0.2}MA_{0.8}PbI_3$. The red lines represent a fit to the data points using a linear function. The slope values are also indicated.

Table S 2: Computed thermal expansion term $\left[\frac{\partial E_g}{\partial T}\right]_{TE}$ from the values of the volumetric thermal expansion coefficient $\alpha_V(T)$, bulk modulus B_0 and gap pressure coefficient $\frac{dE_g}{dP}$ for $FA_{0.2}MA_{0.8}PbI_3$ and different temperatures T. Numbers in parentheses are the error bars.

	$\mathbf{FA_{0.2}MA_{0.8}PbI_3}$				
Phase	T (K)	α_V (10^{-4} K^{-1})	B_0 (GPa)	$\frac{dE_g}{dP}$ (eV/GPa)	$\left[\frac{\partial E_g}{\partial T}\right]_{TE}$ (10^{-4} eV/K)
Ortho	50	1.21(5)	30(6)	-0.043(6)	1.56(20)
	135	1.37(5)	30(6)	0.002(6)	-0.07(12)
	180	1.87(5)	30(6)	0.042(10)	-1.83(30)
	180	1.87(5)	30(6)	0.033(10)	-2.35(30)
Cubic	300	1.69(5)	13(5)	-0.048(8)	1.05(20)
	300	1.69(5)	13(5)	-0.044(10)	0.97(20)
	300	1.69(5)	13(5)	-0.046(3)	1.01(20)
	300	1.69(5)	13(5)	-0.038(3)	0.83(15)

Figure S3 shows the variation with pressure of the gap energy of $FA_{0.4}MA_{0.6}PbI_3$, as obtained from PL line-shape fits of the PL spectra measured at low pressure and at the selected temperatures. The slopes of the linear fits to the data points (red solid lines in Fig. S3) are listed in Table S3 together with the values of B_0 and α_V employed to calculate the thermal expansion term TE.

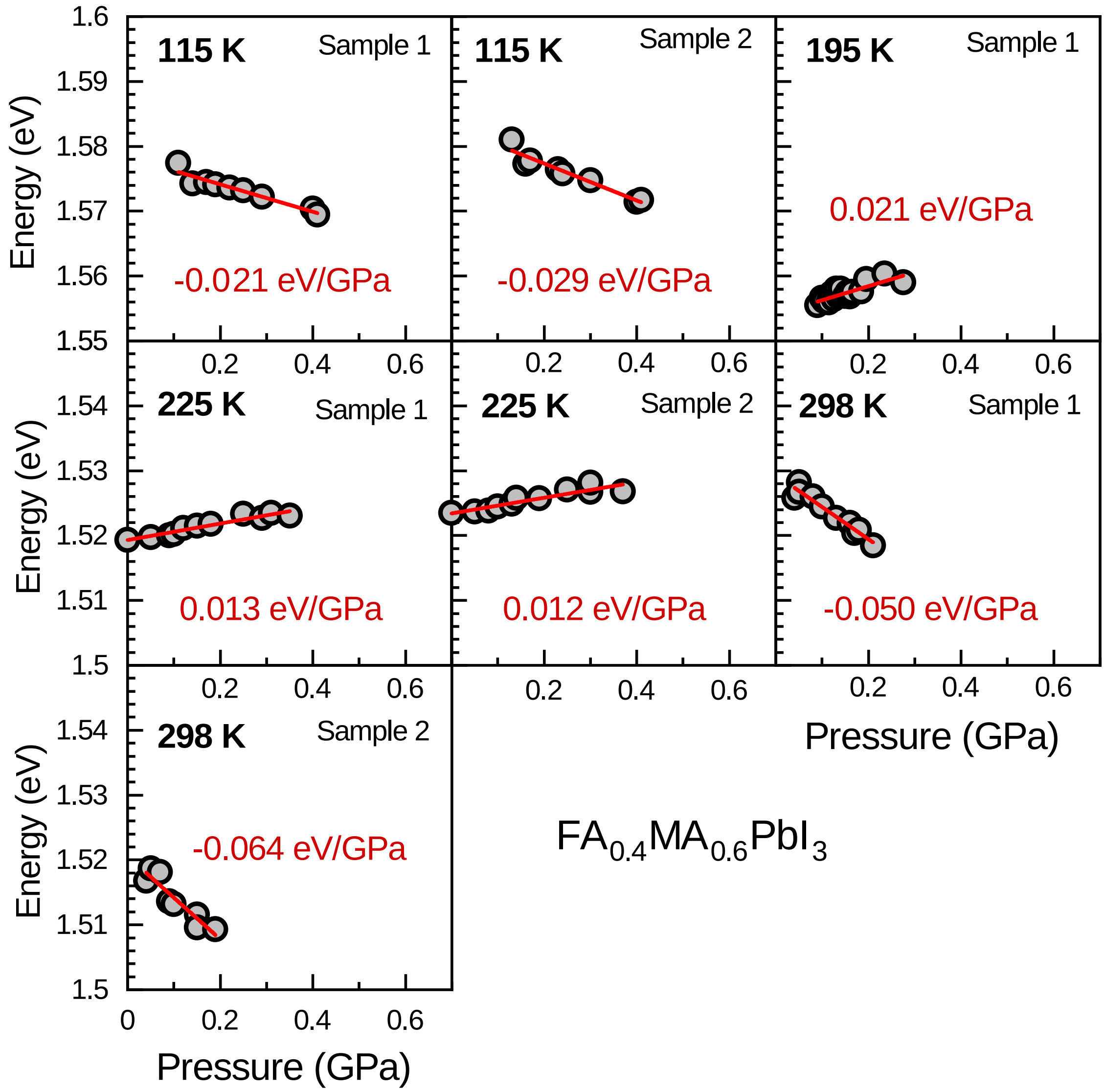

Figure S 3: The energy of the gap extracted from the PL peak maximum (symbols) plotted as a function of pressure and for different temperatures, as indicated, in the case of $FA_{0.4}MA_{0.6}PbI_3$. The red lines represent a fit to the data points using a linear function. The slope values are also indicated.

Table S 3: Computed thermal expansion term $\left[\frac{\partial E_g}{\partial T}\right]_{TE}$ from the values of the volumetric thermal expansion coefficient $\alpha_V(T)$, bulk modulus B_0 and gap pressure coefficient $\frac{dE_g}{dP}$ for $FA_{0.4}MA_{0.6}PbI_3$ and different temperatures T. Numbers in parentheses are the error bars.

	**$FA_{0.4}MA_{0.6}PbI_3$**				
Phase	T (K)	α_V (10^{-4} K^{-1})	B_0 (GPa)	$\frac{dE_g}{dP}$ (eV/GPa)	$\left[\frac{\partial E_g}{\partial T}\right]_{TE}$ (10^{-4} eV/K)
Tetra-III	115	1.90(5)	15(6)	-0.021(5)	0.60(20)
	115	1.90(5)	15(6)	-0.029(5)	0.83(12)
	195	2.30(5)	15(6)	0.021(5)	-0.73(20)
	225	2.30(5)	15(6)	0.013(10)	-0.45(30)
	225	2.30(5)	15(6)	0.012(10)	-0.42(30)
Cubic	298	1.54(5)	12(5)	-0.050(3)	0.92(10)
	298	1.54(5)	12(5)	-0.064(3)	1.18(10)

Figure S4 shows the variation with pressure of the gap energy of $FA_{0.5}MA_{0.5}PbI_3$, as obtained from PL line-shape fits of the PL spectra measured at low pressure and at the selected temperatures. The slopes of the linear fits to the data points (red solid lines in Fig. S4) are listed in Table S4 together with the values of B_0 and α_V employed to calculate the thermal expansion term TE.

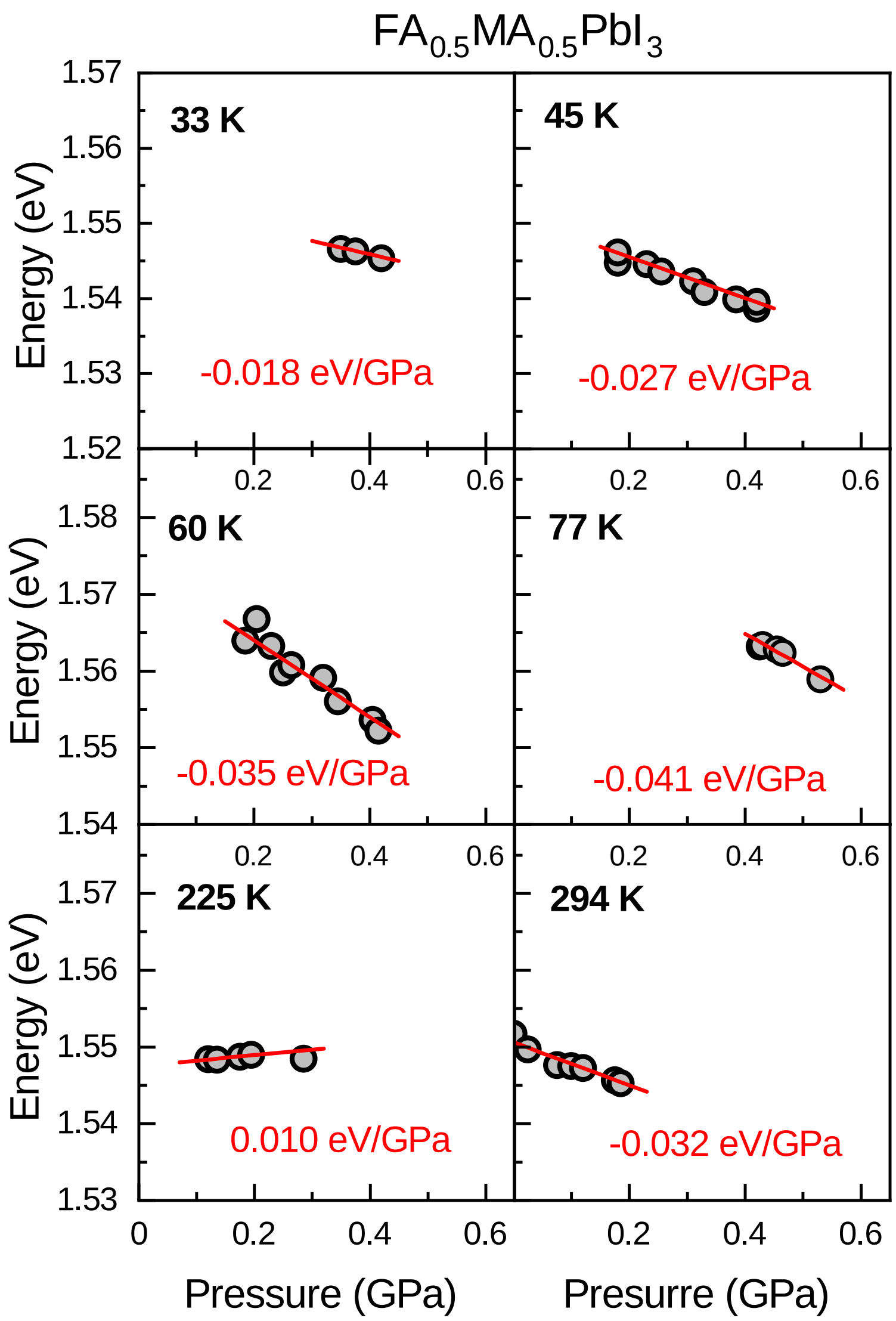

Figure S 4: The energy of the gap extracted from the PL peak maximum (symbols) plotted as a function of pressure and for different temperatures, as indicated, in the case of $FA_{0.5}MA_{0.5}PbI_3$. The red lines represent a fit to the data points using a linear function. The slope values are also indicated.

Table S 4: Computed thermal expansion term $\left[\frac{\partial E_g}{\partial T}\right]_{TE}$ from the values of the volumetric thermal expansion coefficient $\alpha_V(T)$, bulk modulus B_0 and gap pressure coefficient $\frac{dE_g}{dP}$ for $FA_{0.5}MA_{0.5}PbI_3$ and different temperatures T. Numbers in parentheses are the error bars. The asterisk indicates that the data is for the Tetra-III phase of $FAPbI_3$ [25] and the dagger indicates the data is from Ref. [[24]].

	**$FA_{0.5}MA_{0.5}PbI_3$**				
Phase	T (K)	α_V (10^{-4} K^{-1})	B_0 (GPa)	$\frac{dE_g}{dP}$ (eV/GPa)	$\left[\frac{\partial E_g}{\partial T}\right]_{TE}$ (10^{-4} eV/K)
Tetra-III	33	0.32(8)	15(7)	-0.018(15)	0.09(8)
	45	0.99(8)	15(7)	-0.027(15)	0.4 (3)
	60	1.23(8)	15(7)	-0.035(8)	0.65(35)
	77	1.23(8)	15(7)	-0.038(8)	0.7 (4)
	225	1.75(8)*	15(7)	0.010(8)	-0.25(25)
Cubic	294	1.54(5)†	12(5)	-0.032(15)	0.6(3)

Figure S5 shows the variation with pressure of the gap energy of $FA_{0.6}MA_{0.4}PbI_3$, as obtained from PL line-shape fits of the PL spectra measured at low pressure and at the selected temperatures. The slopes of the linear fits to the data points (red solid lines in Fig. S5) are listed in Table S5 together with the values of B_0 and α_V employed to calculate the thermal expansion term TE.

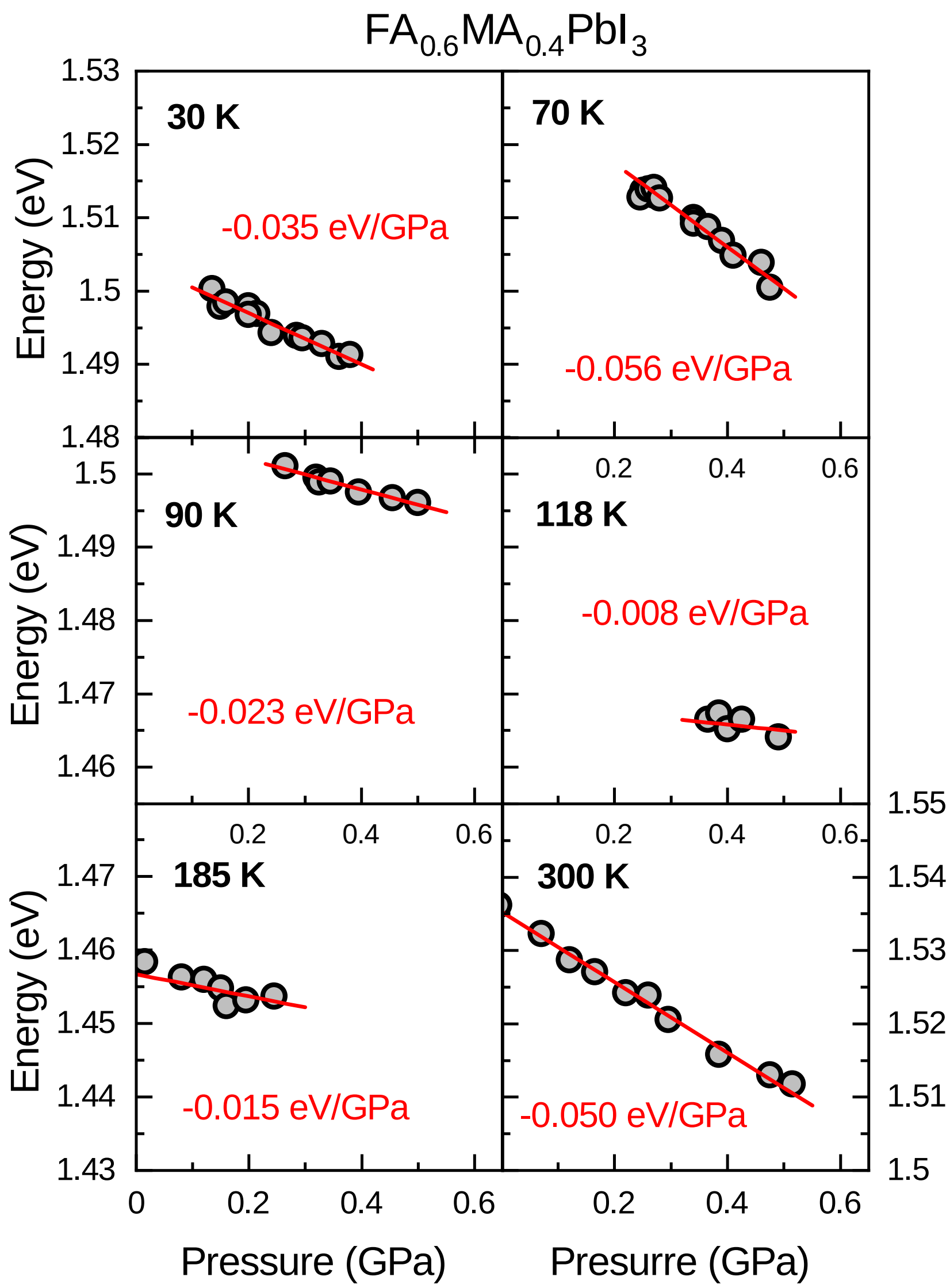

Figure S 5: The energy of the gap extracted from the PL peak maximum (symbols) plotted as a function of pressure and for different temperatures, as indicated, in the case of $FA_{0.6}MA_{0.4}PbI_3$. The red lines represent a fit to the data points using a linear function. The slope values are also indicated.

Table S 5: Computed thermal expansion term $\left[\frac{\partial E_g}{\partial T}\right]_{TE}$ from the values of the volumetric thermal expansion coefficient $\alpha_V(T)$, bulk modulus B_0 and gap pressure coefficient $\frac{dE_g}{dP}$ for $FA_{0.6}MA_{0.4}PbI_3$ and different temperatures T. Numbers in parentheses are the error bars.

	$\mathbf{FA_{0.6}MA_{0.4}PbI_3}$				
Phase	T (K)	α_V (10^{-4} K^{-1})	B_0 (GPa)	$\frac{dE_g}{dP}$ (eV/GPa)	$\left[\frac{\partial E_g}{\partial T}\right]_{TE}$ (10^{-4} eV/K)
Tetra-III	30	0.99(8)	15(7)	-0.035(4)	0.5(1)
	70	1.23(8)	15(7)	-0.056(10)	1.0(3)
	90	1.75(8)	15(7)	-0.023(6)	0.6(2)
	118	1.75(8)	15(7)	-0.008(6)	0.2(2)
Tetra-II	185	1.90(6)	12(7)	-0.015(5)	0.35(20)
	207	1.90(6)	12(7)	-0.003(5)	0.07(6)
Cubic	300	1.54(5)	12(5)	-0.060(10)	1.1(2)
	300	1.54(5)	12(5)	-0.050(5)	0.9(1)
	300	1.54(5)	12(5)	-0.054(8)	1.0(2)

Figure S6 shows the variation with pressure of the gap energy of $FA_{0.8}MA_{0.2}PbI_3$, as obtained from PL line-shape fits of the PL spectra measured at low pressure and at the selected temperatures. The slopes of the linear fits to the data points (red solid lines in Fig. S6) are listed in Table S6 together with the values of B_0 and α_V employed to calculate the thermal expansion term TE.

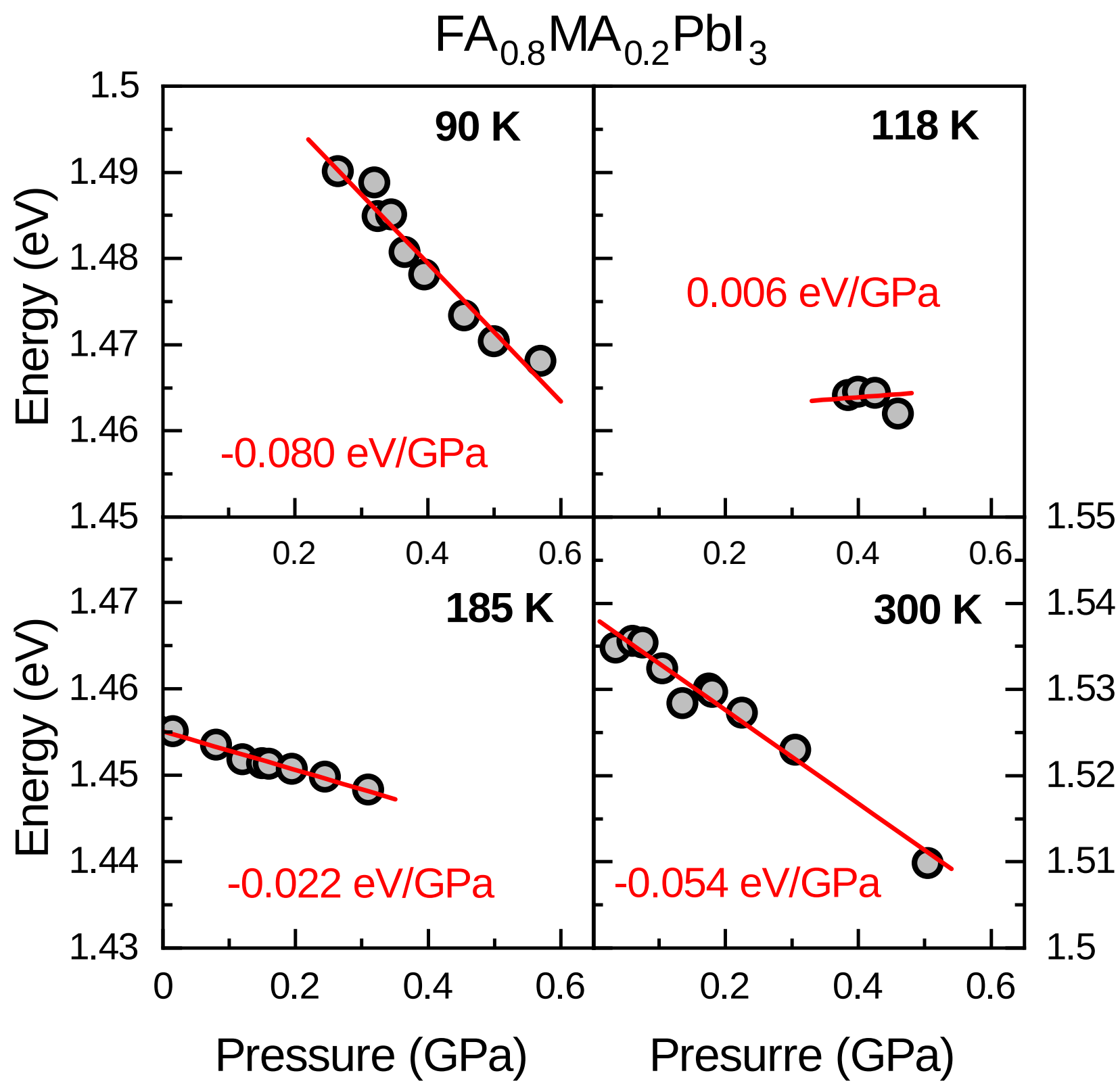

Figure S 6: The energy of the gap extracted from the PL peak maximum (symbols) plotted as a function of pressure and for different temperatures, as indicated, in the case of $FA_{0.8}MA_{0.2}PbI_3$. The red lines represent a fit to the data points using a linear function. The slope values are also indicated.

Table S 6: Computed thermal expansion term $\left[\frac{\partial E_g}{\partial T}\right]_{TE}$ from the values of the volumetric thermal expansion coefficient $\alpha_V(T)$, bulk modulus B_0 and gap pressure coefficient $\frac{dE_g}{dP}$ for $FA_{0.8}MA_{0.2}PbI_3$ and different temperatures T. Numbers in parentheses are the error bars.

	**$FA_{0.8}MA_{0.2}PbI_3$**				
Phase	T (K)	α_V (10^{-4} K^{-1})	B_0 (GPa)	$\frac{dE_g}{dP}$ (eV/GPa)	$\left[\frac{\partial E_g}{\partial T}\right]_{TE}$ (10^{-4} eV/K)
Tetra-III	90	1.75(8)	15(7)	-0.080(15)	2.1 (5)
	118	1.90(8)	12(7)	0.006(8)	-0.14(3)
Tetra-II	185	1.90(6)	12(7)	-0.022(5)	0.5 (2)
Cubic	300	1.08(5)	12(5)	-0.054(5)	0.7(1)

Figure S7 shows the variation with pressure of the gap energy of $FA_{0.9}MA_{0.1}PbI_3$, as obtained

from PL line-shape fits of the PL spectra measured at low pressure and at the selected temperatures. The slopes of the linear fits to the data points (red solid lines in Fig. S7) are listed in Table S7 together with the values of B_0 and α_V employed to calculate the thermal expansion term TE.

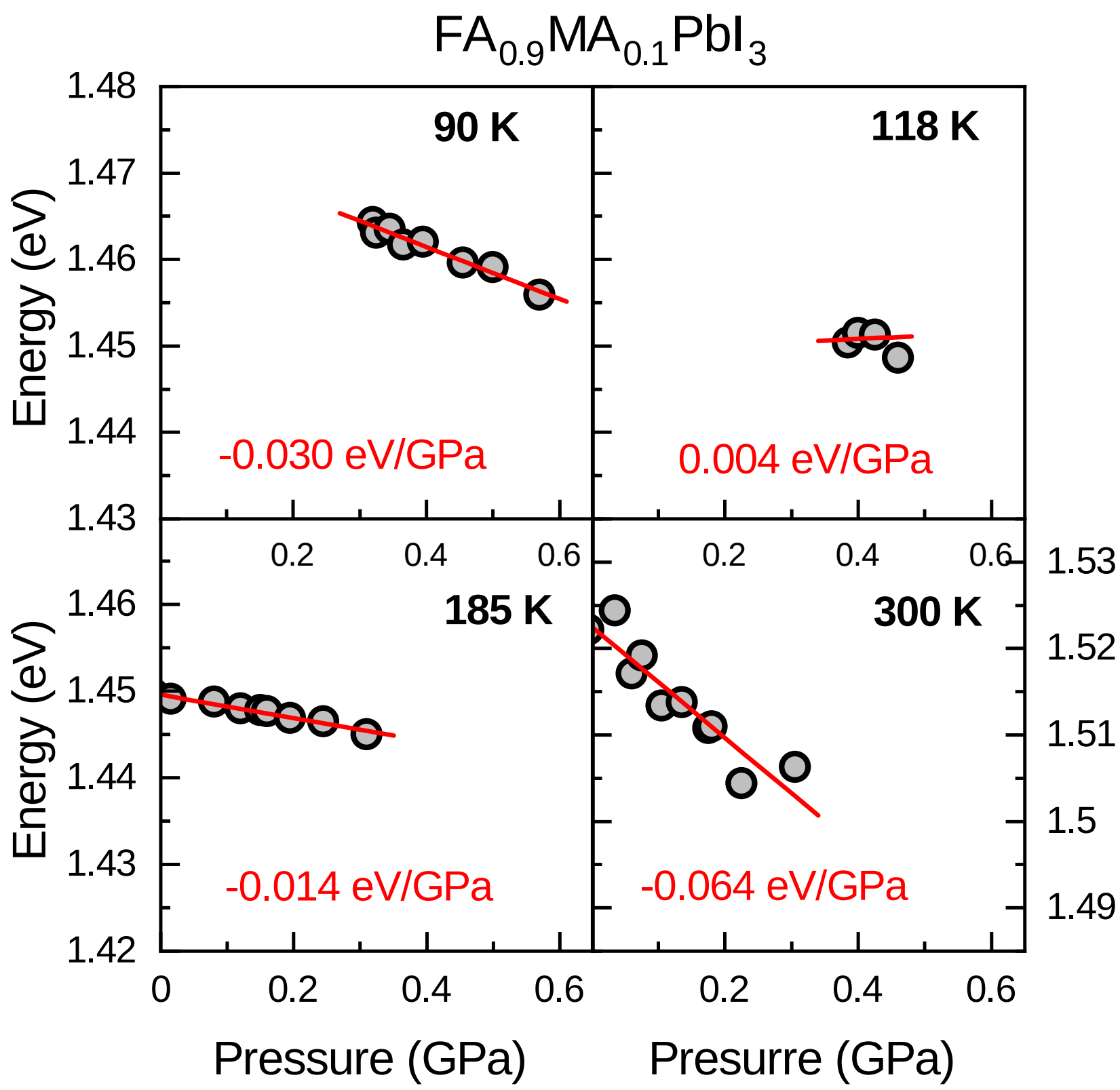

Figure S 7: The energy of the gap extracted from the PL peak maximum (symbols) plotted as a function of pressure and for different temperatures, as indicated, in the case of $FA_{0.9}MA_{0.1}PbI_3$. The red lines represent a fit to the data points using a linear function. The slope values are also indicated.

Table S 7: Computed thermal expansion term $\left[\frac{\partial E_g}{\partial T}\right]_{TE}$ from the values of the volumetric thermal expansion coefficient $\alpha_V(T)$, bulk modulus B_0 and gap pressure coefficient $\frac{dE_g}{dP}$ for $FA_{0.9}MA_{0.1}PbI_3$ and different temperatures T. Numbers in parentheses are the error bars.

	**$FA_{0.9}MA_{0.1}PbI_3$**				
Phase	T (K)	α_V (10^{-4} K^{-1})	B_0 (GPa)	$\frac{dE_g}{dP}$ (eV/GPa)	$\left[\frac{\partial E_g}{\partial T}\right]_{TE}$ (10^{-4} eV/K)
Tetra-III	90	1.75(8)	15(7)	-0.030(8)	0.8 (2)
	118	1.90(8)	12(7)	0.004(10)	-0.09(2)
Tetra-II	185	1.90(6)	12(7)	-0.014(5)	0.3 (1)
Cubic	300	1.08(5)	12(5)	-0.064(6)	0.8(1)

Note ♯5: The electron-phonon (EP) coupling term

Gopalan *et al.*[17] derived an expression for the energy shift of the gap induced by temperature, through electron-phonon interaction, of any electronic state $E_{n\mathbf{k}}$ with band index n and wavevector $\mathbf{k}$, for which all phonon modes of the branch j, wavevector $\mathbf{q}$ and frequency $\omega_{j\mathbf{q}}$ contribute:

$$\Delta E_{n\mathbf{k}}(T) = \sum_{j\mathbf{q}} \frac{\partial E_{n\mathbf{k}}}{\partial n_{j\mathbf{q}}} \left(n_{j\mathbf{q}}(T) + \frac{1}{2} \right), \tag{4}$$

where $n_{j\mathbf{q}} = \left(e^{\beta \hbar \omega_{j\mathbf{q}}} - 1\right)^{-1}$ is the Bose-Einstein phonon occupation factor with $\beta = \frac{1}{k_B T}$. Importantly, the complex interaction coefficients $\frac{\partial E_{n\mathbf{k}}}{\partial n_{j\mathbf{q}}}$ contribute *both* to the energy shift of the bands as well as to a lifetime broadening of the electronic states (linewidths). Obviously, the sign of the renormalization of a gap is thus determined by the difference in magnitude and sign of the respective energy shift of valence and conduction band.

By invoking energy conservation, the summation in Eq. (4) transforms into an integral over the

phonon frequencies:[17]

$$\Delta E_{n\mathbf{k}}(T) = \int_0^\infty d\omega \cdot g^2F(n,\mathbf{k},\omega)\cdot\left(n_{j\mathbf{q}}(T)+\frac{1}{2}\right)$$
$$g^2F(n,\mathbf{k},\omega) = \sum_{j\mathbf{q}} \frac{\partial E_{n\mathbf{k}}}{\partial n_{j\mathbf{q}}}\delta(\omega-\omega_{j\mathbf{q}}). \tag{5}$$

The function $g^2F(n,\mathbf{k},\omega)$ is the so-called electron-phonon spectral function and is essentially the phonon density of states (DOS) appropriately weighted by electron-phonon matrix elements. As such, the spectral function is temperature independent, which means that the temperature dependence of the electron-phonon contribution to the gap shift arises solely from the Bose-Einstein occupation factor $n_{j\mathbf{q}}(T)$.

Equations. (4) and (5) indicate that the main contributions to the electron-phonon renormalization of the gap arise from peaks in the DOS. In fact, this is at the origin of the Einstein-oscillator model introduced by Cardona and coworkers,[18,30,31] which approximate the $\frac{\partial E_{n\mathbf{k}}}{\partial n_{j\mathbf{q}}}$ coefficients by effective electron-phonon interaction parameters A_i for phonons with average frequency ω_i, inferred from the peaks in the phonon DOS. The EP correction to the gap then reads

$$[\Delta E_g(T)]_{EP} = \sum_i A_i\cdot\left(n_B(\omega_i,T)+\frac{1}{2}\right), \tag{6}$$

where n_B again stands for the Bose-Einstein occupation factor of the effective mode with frequency ω_i. The analytical expression for the EP term is simply obtained by taking the first derivative of the Bose-Einstein occupation number over temperature as:

$$\left[\frac{\partial E_g}{\partial T}\right]_{EP} = \sum_i A_i\cdot\frac{\partial n_B(\omega_i,T)}{\partial T}$$
$$= \sum_i \frac{A_i}{4T}\cdot\frac{\hbar\omega_i}{k_BT}\cdot\frac{1}{sinh^2\left(\frac{\hbar\omega_i}{2k_BT}\right)}. \tag{7}$$

Here $n_B(\omega_i,T)$ is the Bose-Einstein phonon occupation factor of the ith oscillator. The impor-

tance of the previous equation is that allows for computing the renormalization of the gap at zero temperature, where the correction to the gap energy is given by the effect of the zero-point vibrations. Using the Einstein oscillator model the zero-point correction (ZPC) to the gap can be readily obtained by considering the limit to zero temperature of the expression in Eq. (6):

$$[\Delta E_g(0K)]_{EP} = \frac{1}{2} \cdot \sum_{i=1}^{n} A_i, \tag{8}$$

where $n = 1$ or 2 for no or finite FA content, as shown further below.

To obtain the EP term, it is convenient to perform least-squares fits using the expression derived for the first derivative of the curves gap-energy-vs-temperature. The resulting first-derivative data points (closed symbols) are shown in Figs. 3a-c of the main manuscript for x=0, 0.2 and 0.8, respectively, and in the following for the rest of FA concentrations that were studied jointly as a function of pressure and temperature. We first consider the case of the archetypal perovskite $MAPbI_3$ for which an equal footing of thermal expansion and electron-phonon interaction effects on the temperature renormalization of the gap has been obtained at room temperature.[32] To obtain the EP term (or terms) by fitting, *only* the data points within the temperature range of stability of the different crystal phases were considered, provided the TE term was also determined for the corresponding temperature range. These are the blue dots in Fig. 8 (Fig. 3a of the main manuscript) and which correspond to the TE term values resulting from Eq. (3) and tabulated in Table 1. For the cubic and tetragonal-I phases the blue dot-dashed lines indicate that the TE term is temperature independent, coinciding with the linearity of the gap temperature dependence for these phases. The contribution from electron-phonon interaction is calculated using the function of Eq. (7). However, for the orthorhombic phase the temperature dependence of the gap is clearly non-linear, which strongly speaks for a TE term that is temperature dependent. For this reason we have to pursue the measurements of the TE term at different cryogenic temperatures with the outcome tabulated in Table 1. Thus, for the fits to determine the EP term, the TE contribution will be represented by a polynomial function with fixed coefficients that inter/extrapolate the measured data points. This function is forced to be zero at the absolute zero, since $\alpha_V(T=0) \equiv 0$. In the particular case of the or-

thorhombic phase of $MAPbI_3$ the TE term function reads: $(a_0 + a_1 T + a_2 T^2) \cdot \left(\frac{I_0 \cdot \omega_0}{4 k_B T^2 sinh^2\left(\frac{\omega_0}{2k_B T}\right)} \right)$ with $a_0 = 0.575$ eV/K, $a_1 = -1.224 \cdot 10^{-3}$ eV/K^2, $a_2 = -2.97 \cdot 10^{-5}$ eV/K^3, $\omega_0 = 1$ meV, and $I_0 = 2.9$ eV. As demonstrated for the tetragonal-I phase of $MAPbI_3$,[32] a single Einstein oscillator is sufficient for the proper description of the EP term for the other phases too. The function of Eq. (7) together with the corresponding contribution from TE were fitted, piecewise for each phase, to the data points of Fig. 3a of the main manuscript. The resulting oscillator frequencies and amplitudes are listed in Table S8. The solid black curves and the dashed red curves in Fig. 8 (Fig. 3a) represent the resulting total rate of gap renormalization per Kelvin and the EP contributions to it, respectively.

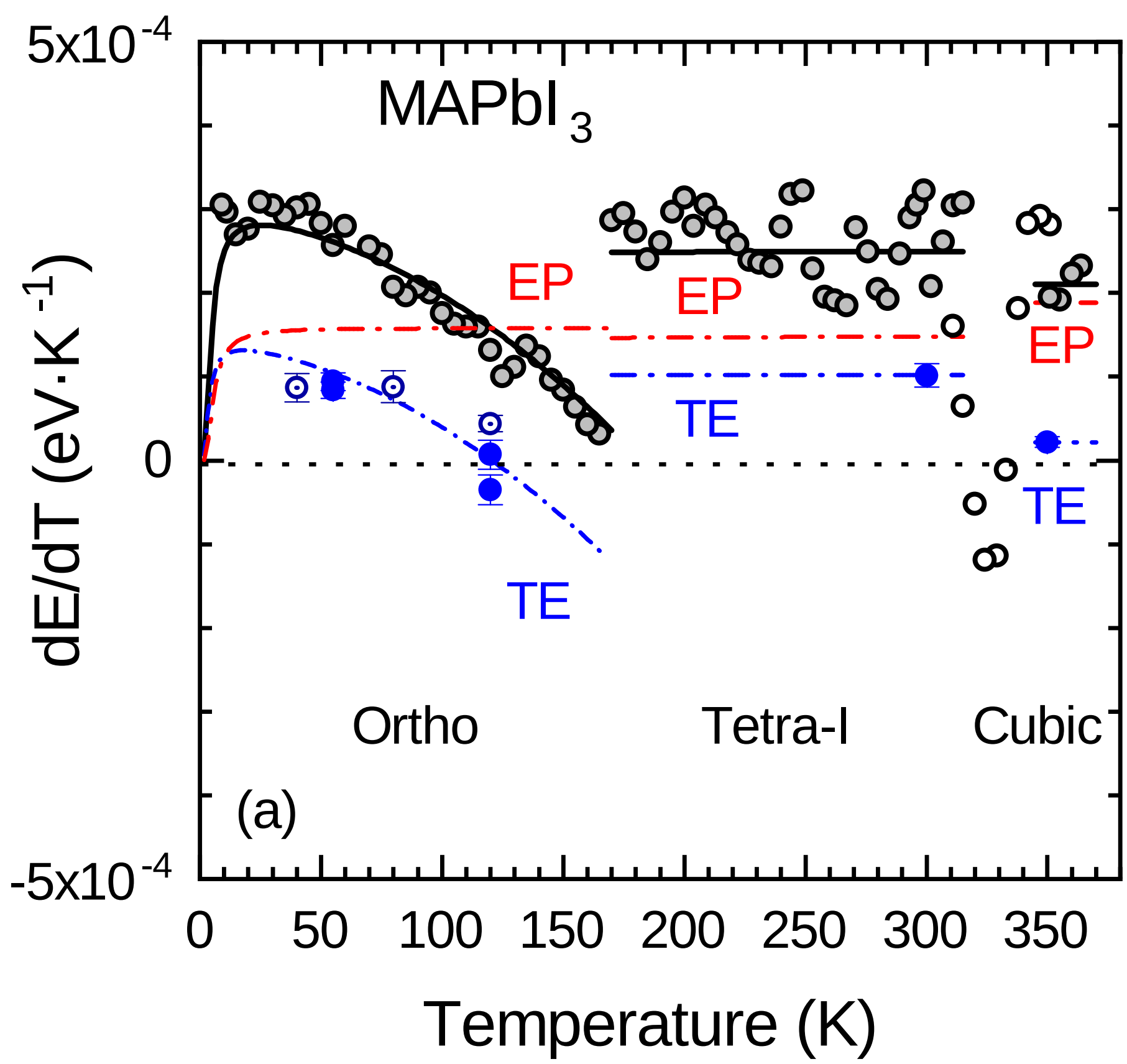

Figure S 8: Numerically calculated first derivative of the PL peak energy with respect to temperature (black-grey symbols) for $MAPbI_3$. The solid black lines represent a fit only to the data points in the temperature range of stability of the different crystal phases, corresponding to the sum of the contribution of thermal expansion (TE, blue dot-dashed curve and symbols) and electron-phonon interaction (EP, red dot-dashed curve).

Table S 8: Einstein-oscillator parameters corresponding to the effective amplitude A_i and frequency ω_i describing the contribution from electron-phonon interaction $\left[\frac{\partial E_g}{\partial T}\right]_{EP}$ to the renormalization of the band gap in $MAPbI_3$. Numbers in parentheses are the error bars (uncertainty of the last digits) of the adjustable fitting parameters. Values marked with an asterisk are from Ref. [32]. Also listed are the value of the calculated zero-point corrections (ZPC) according to Eq. (8) and the temperature range in which the fit is valid.

	**$MAPbI_3$**				
Phase	Einstein osc.	A_i (meV)	$\hbar\omega_i$ (meV)	ZPC (meV)	T fitting range (K)
Cubic	Osc. ♯1	13(1)	6		>345
Tetra-I	Osc. ♯1	10(1)*	5.8(6)*		170 - 315
Ortho	Osc. ♯1	2.7(5)	1.5(10)	1 (2)	<165

For the orthorhombic phase of $FA_{0.2}MA_{0.8}PbI_3$ the TE term function is a third-order polynomial $a_0 + a_1T + a_2T^2 + a_3T^3$ with $a_0 = 0$, $a_1 = 6.15 \cdot 10^{-6}$ eV/K^2, $a_2 = -6.4 \cdot 10^{-8}$ eV/K^3, and $a_3 = 1.33 \cdot 10^{-10}$ eV/K^4. Again, for the cubic and tetragonal-I phases a single Einstein oscillator is sufficient to describe the EP term, whereas for the low-temperature orthorhombic phase an anomalous electron-phonon coupling sets in that is accounted for with a second Einstein oscillator with negative amplitude. The results of the least-square fitting to the first-derivative data points of Fig. 9 (Fig. 3b of the main manuscript) corresponding to the frequency and amplitude of the different Einstein oscillators are listed in Table S9.

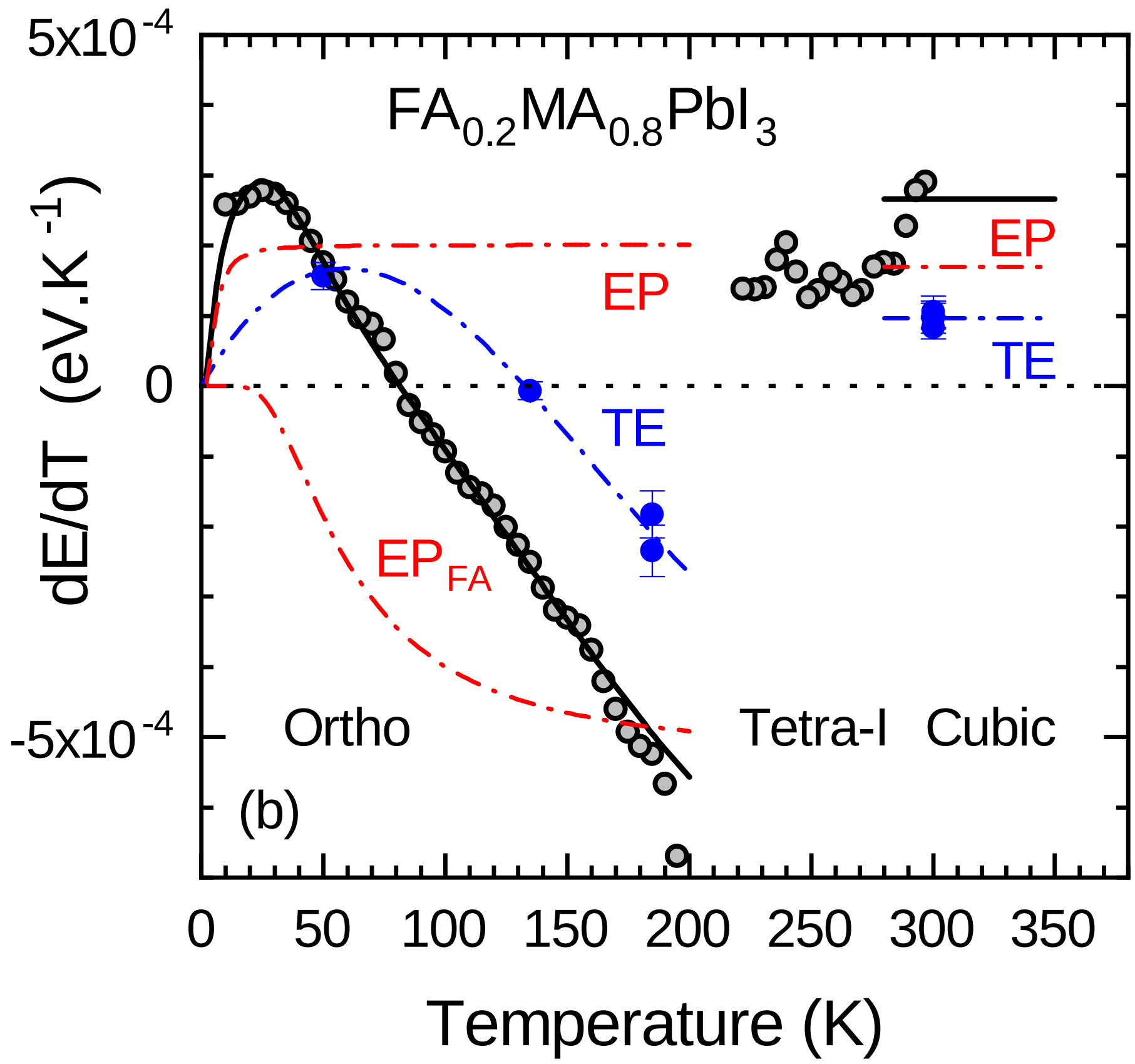

Figure S 9: Numerically calculated first derivative of the PL peak energy with respect to temperature (black-grey symbols) for $FA_{0.2}MA_{0.8}PbI_3$ mixed-cation perovskite. The solid black lines represent a fit only to the data points in the temperature range of stability of the different crystal phases, corresponding to the sum of the contribution of thermal expansion (TE, blue dot-dashed curve and symbols) and electron-phonon interaction (EP, red dot-dashed curve). The curve labeled EP_{FA} represents the additional Einstein oscillator introduced to account for the anomalous electron-phonon interaction due to coupling to FA rattler modes.

Table S 9: Einstein-oscillator parameters corresponding to the effective amplitude A_i and frequency ω_i describing the contribution from electron-phonon interaction $\left[\frac{\partial E_g}{\partial T}\right]_{EP}$ to the renormalization of the band gap in $FA_{0.2}MA_{0.8}PbI_3$. Numbers in parentheses are the error bars (uncertainty of the last digits) of the adjustable fitting parameters. Also listed are the value of the calculated zero-point corrections (ZPC) according to Eq. (8) and the temperature range in which the fit is valid.

	$\mathbf{FA_{0.2}MA_{0.8}PbI_3}$				
Phase	Einstein osc.	A_i (meV)	$\hbar\omega_i$ (meV)	ZPC (meV)	T fitting range (K)
Cubic	Osc. ♯1	13(1)	6		>285
Tetra-I					215 - 280
Ortho	Osc. ♯1	3.5(5)	1.5	-45(15)	<170
	Osc. ♯2	-98(10)	16(1)		

For the tetragonal-III phase of $FA_{0.4}MA_{0.6}PbI_3$ the TE term function is also a third-order polynomial $a_0 + a_1T + a_2T^2 + a_3T^3$ with $a_0 = 0$, $a_1 = 7.34 \cdot 10^{-6}$ eV/K^2, $a_2 = -8.63 \cdot 10^{-8}$ eV/K^3, and $a_3 = 2.35 \cdot 10^{-10}$ eV/K^4. Again, for the cubic phase a single Einstein oscillator is sufficient to describe the EP term, whereas for the low-temperature tetragonal-III phase the second Einstein oscillator corresponding to the anomalous electron-phonon coupling also has negative amplitude. The results of the least-square fitting to the first-derivative data points of Fig. 10 corresponding to the frequency and amplitude of the different Einstein oscillators are listed in Table S10. We note that for the remaining compositions, including this one, the frequency of the FA rattler depends slightly on the temperature range considered for the least-squares fits. Typically, the smaller the range, the smaller the frequency. We thus tabulated both results but show the one corresponding to the extended T range in the corresponding figures.

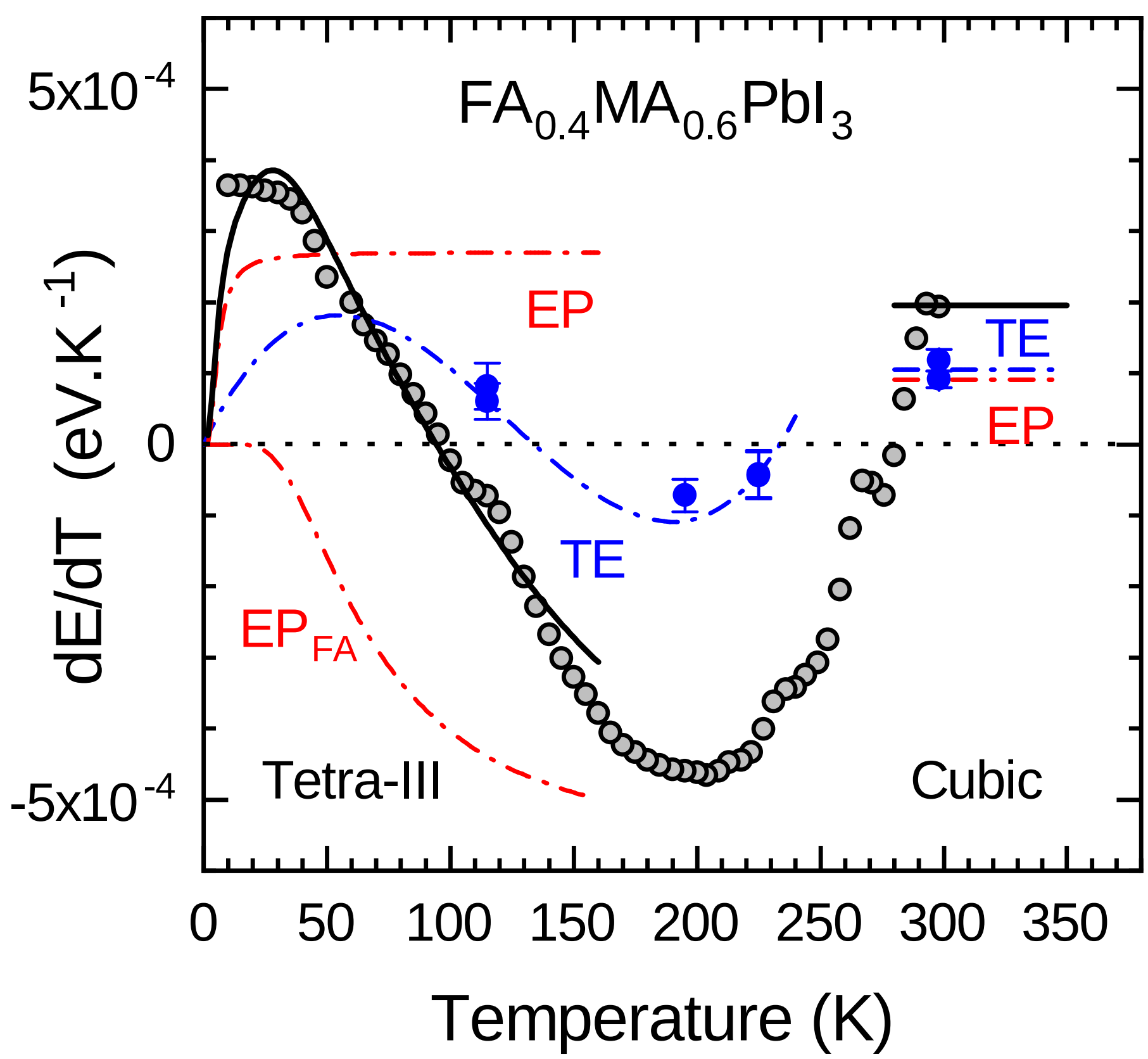

Figure S 10: Numerically calculated first derivative of the PL peak energy with respect to temperature (black-grey symbols) for $FA_{0.4}MA_{0.6}PbI_3$ mixed-cation perovskite. The solid black lines represent a fit only to the data points in the temperature range of stability of the different crystal phases, corresponding to the sum of the contribution of thermal expansion (TE, blue dot-dashed curve and symbols) and electron-phonon interaction (EP, red dot-dashed curve). The curve labeled EP_{FA} represents the additional Einstein oscillator introduced to account for the anomalous electron-phonon interaction due to coupling to FA rattler modes.

Table S 10: Einstein-oscillator parameters corresponding to the effective amplitude A_i and frequency ω_i describing the contribution from electron-phonon interaction $\left[\frac{\partial E_g}{\partial T}\right]_{EP}$ to the renormalization of the band gap in $FA_{0.4}MA_{0.6}PbI_3$. Numbers in parentheses are the error bars (uncertainty of the last digits) of the adjustable fitting parameters. Also listed are the value of the calculated zero-point corrections (ZPC) according to Eq. (8) and the temperature range in which the fit is valid.

	**$FA_{0.4}MA_{0.6}PbI_3$**				
Phase	Einstein osc.	A_i (meV)	$\hbar\omega_i$ (meV)	ZPC (meV)	T fitting range (K)
Cubic	Osc. $\sharp$1	6(1)	6		>295
Tetra-III	Osc. $\sharp$1	6(1)	1.5	-40(15)	<130
	Osc. $\sharp$2	-85(10)	14(1)		
	Osc. $\sharp$1	5(1)	1.5	-55(20)	<170
	Osc. $\sharp$2	-120(10)	18(3)		

For the tetragonal-III phase of $FA_{0.5}MA_{0.5}PbI_3$ the TE term function is now a fourth-order polynomial $a_0 + a_1 T + a_2 T^2 + a_3 T^3 + a_4 T^4$ with $a_0 = 0$, $a_1 = 1.22 \cdot 10^{-7}$ eV/K^2, $a_2 = 3.67 \cdot 10^{-8}$ eV/K^3, $a_3 = -4.37 \cdot 10^{-10}$, and $a_4 = 1.2 \cdot 10^{-12}$ eV/K^5. Again, for the cubic phase a single Einstein oscillator is sufficient to describe the EP term, whereas for the low-temperature tetragonal-III phase the second Einstein oscillator corresponding to the anomalous electron-phonon coupling also has negative amplitude. The results of the least-square fitting to the first-derivative data points of Fig. 11 corresponding to the frequency and amplitude of the different Einstein oscillators are listed in Table S11.

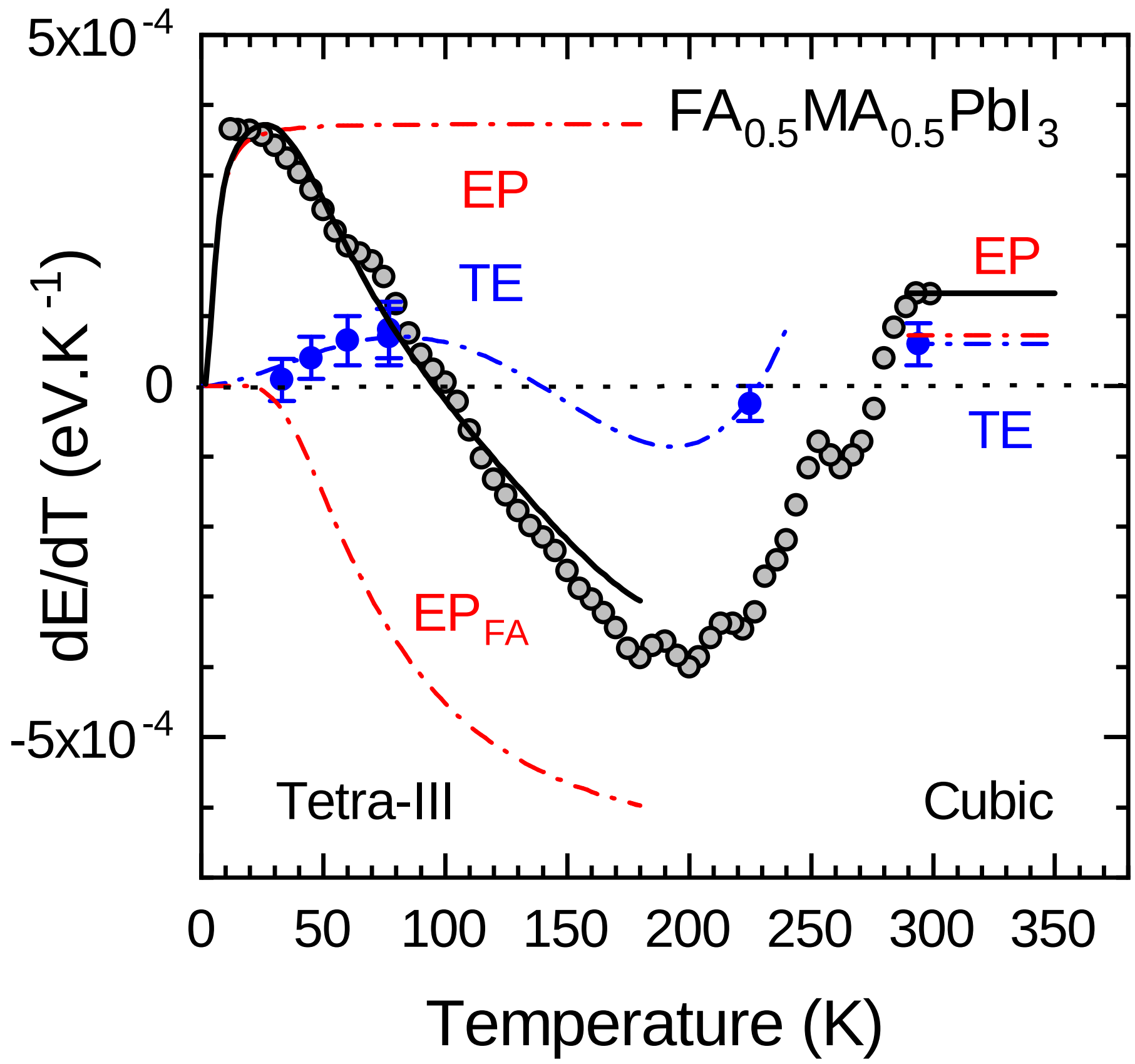

Figure S 11: Numerically calculated first derivative of the PL peak energy with respect to temperature (black-grey symbols) for $FA_{0.5}MA_{0.5}PbI_3$ mixed-cation perovskite. The solid black lines represent a fit only to the data points in the temperature range of stability of the different crystal phases, corresponding to the sum of the contribution of thermal expansion (TE, blue dot-dashed curve and symbols) and electron-phonon interaction (EP, red dot-dashed curve). The curve labeled EP_{FA} represents the additional Einstein oscillator introduced to account for the anomalous electron-phonon interaction due to coupling to FA rattler modes.

Table S 11: Einstein-oscillator parameters corresponding to the effective amplitude A_i and frequency ω_i describing the contribution from electron-phonon interaction $\left[\frac{\partial E_g}{\partial T}\right]_{EP}$ to the renormalization of the band gap in $FA_{0.5}MA_{0.5}PbI_3$. Numbers in parentheses are the error bars (uncertainty of the last digits) of the adjustable fitting parameters. Also listed are the value of the calculated zero-point corrections (ZPC) according to Eq. (8) and the temperature range in which the fit is valid.

	**$FA_{0.5}MA_{0.5}PbI_3$**				
Phase	Einstein osc.	A_i (meV)	$\hbar\omega_i$ (meV)	ZPC (meV)	T fitting range (K)
Cubic	Osc. ♯1	5(1)	6		>290
Tetra-III	Osc. ♯1	7(1)	1.5	-65(15)	<120
	Osc. ♯2	-135(10)	18(2)		
	Osc. ♯1	6(1)	1.5	-75(20)	<180
	Osc. ♯2	-150(10)	19(3)		

For the tetragonal-III phase of $FA_{0.6}MA_{0.4}PbI_3$ the TE term function is a third-order polynomial $a_0 + a_1 T + a_2 T^2 + a_3 T^3$ with $a_0 = 0$, $a_1 = 3.82 \cdot 10^{-6}$ eV/K^2, $a_2 = -4.45 \cdot 10^{-8}$ eV/K^3, and $a_3 = 1.2 \cdot 10^{-10}$. Again, for the cubic phase a single Einstein oscillator is sufficient to describe the EP term, whereas for the low-temperature tetragonal-III phase the second Einstein oscillator corresponding to the anomalous electron-phonon coupling also has negative amplitude. The results of the least-square fitting to the first-derivative data points of Fig. 12 corresponding to the frequency and amplitude of the different Einstein oscillators are listed in Table S12.

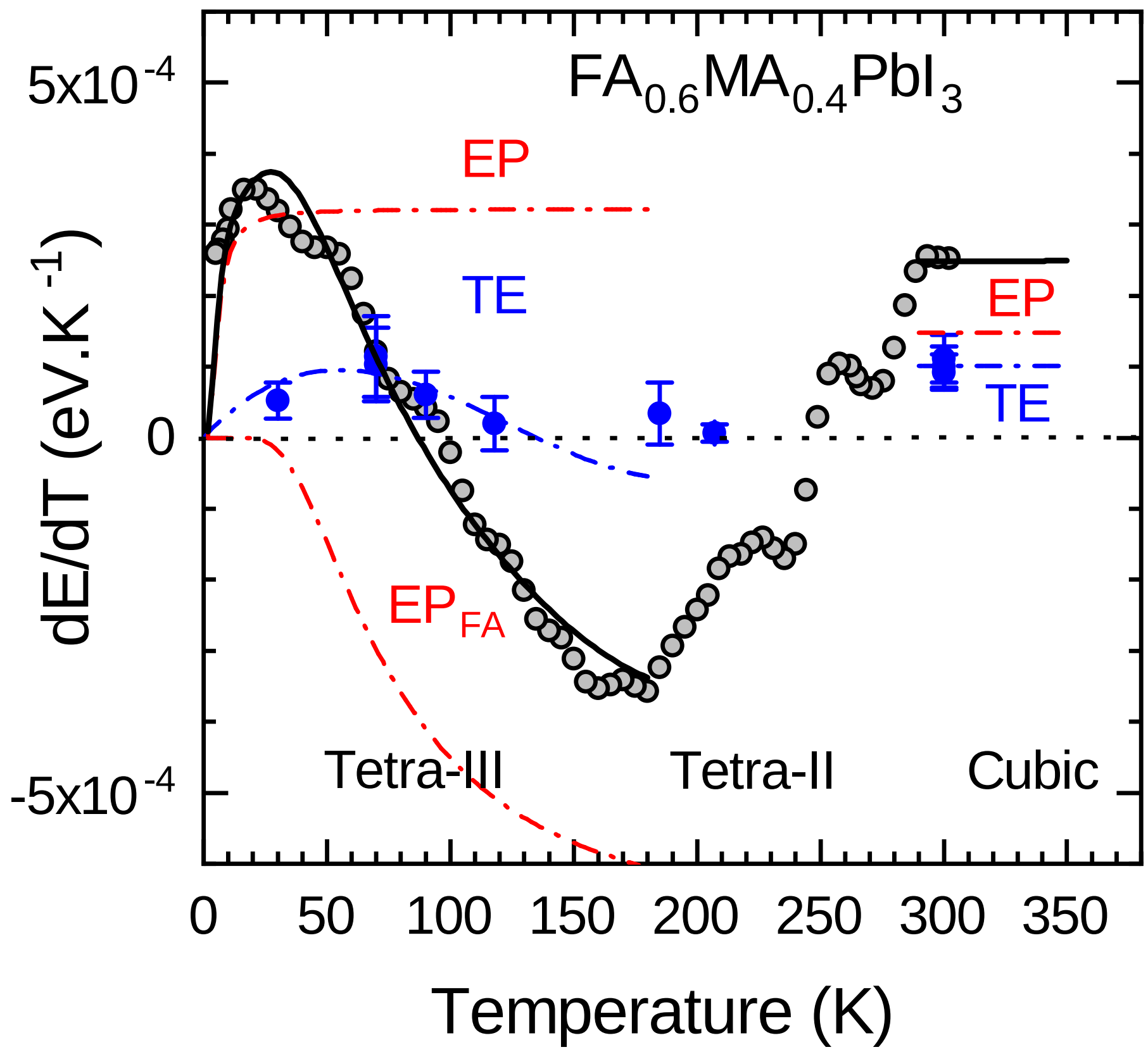

Figure S 12: Numerically calculated first derivative of the PL peak energy with respect to temperature (black-grey symbols) for $FA_{0.6}MA_{0.4}PbI_3$ mixed-cation perovskite. The solid black lines represent a fit only to the data points in the temperature range of stability of the different crystal phases, corresponding to the sum of the contribution of thermal expansion (TE, blue dot-dashed curve and symbols) and electron-phonon interaction (EP, red dot-dashed curve). The curve labeled EP_{FA} represents the additional Einstein oscillator introduced to account for the anomalous electron-phonon interaction due to coupling to FA rattler modes.

Table S 12: Einstein-oscillator parameters corresponding to the effective amplitude A_i and frequency ω_i describing the contribution from electron-phonon interaction $\left[\frac{\partial E_g}{\partial T}\right]_{EP}$ to the renormalization of the band gap in $FA_{0.6}MA_{0.4}PbI_3$. Numbers in parentheses are the error bars (uncertainty of the last digits) of the adjustable fitting parameters. Also listed are the value of the calculated zero-point corrections (ZPC) according to Eq. (8) and the temperature range in which the fit is valid.

	**$FA_{0.6}MA_{0.4}PbI_3$**				
Phase	Einstein osc.	A_i (meV)	$\hbar\omega_i$ (meV)	ZPC (meV)	T fitting range (K)
Cubic	Osc. ♯1	10(1)	6		>290
Tetra-II					200 - 250
Tetra-III	Osc. ♯1	6(1)	1.5	-60(15)	<125
	Osc. ♯2	-125(10)	18(3)		
	Osc. ♯1	6(1)	1.5	-75(20)	<180
	Osc. ♯2	-160(10)	20(3)		

For the tetragonal-III phase of $FA_{0.8}MA_{0.2}PbI_3$ the TE term function is a third-order polynomial $a_0 + a_1T + a_2T^2 + a_3T^3$ with $a_0 = 0$, $a_1 = 1.04 \cdot 10^{-5}$ eV/K^2, $a_2 = -1.29 \cdot 10^{-7}$ eV/K^3, and $a_3 = 3.7 \cdot 10^{-10}$. Again, for the cubic and tetragonal-II phases a single Einstein oscillator is sufficient to describe the EP term, whereas for the low-temperature tetragonal-III phase the second Einstein oscillator corresponding to the anomalous electron-phonon coupling also has negative amplitude. The results of the least-square fitting to the first-derivative data points of Fig. 13 (Fig. 3c of the main manuscript) corresponding to the frequency and amplitude of the different Einstein oscillators are listed in Table S13.

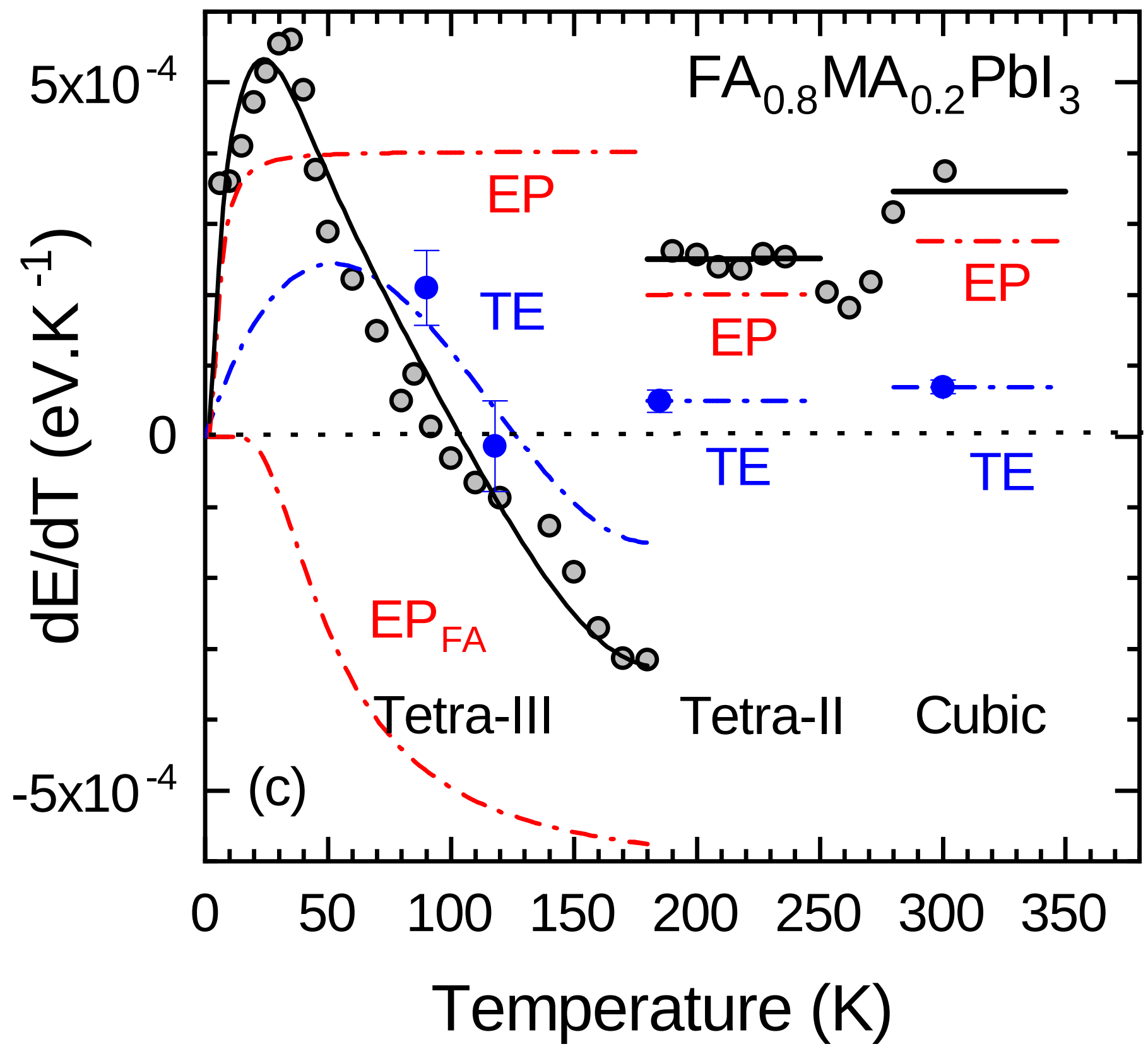

Figure S 13: Numerically calculated first derivative of the PL peak energy with respect to temperature (black-grey symbols) for $FA_{0.8}MA_{0.2}PbI_3$ mixed-cation perovskite. The solid black lines represent a fit only to the data points in the temperature range of stability of the different crystal phases, corresponding to the sum of the contribution of thermal expansion (TE, blue dot-dashed curve and symbols) and electron-phonon interaction (EP, red dot-dashed curve). The curve labeled EP_{FA} represents the additional Einstein oscillator introduced to account for the anomalous electron-phonon interaction due to coupling to FA rattler modes.

Table S 13: Einstein-oscillator parameters corresponding to the effective amplitude A_i and frequency ω_i describing the contribution from electron-phonon interaction $\left[\frac{\partial E_g}{\partial T}\right]_{EP}$ to the renormalization of the band gap in $FA_{0.8}MA_{0.2}PbI_3$. Numbers in parentheses are the error bars (uncertainty of the last digits) of the adjustable fitting parameters. Also listed are the value of the calculated zero-point corrections (ZPC) according to Eq. (8) and the temperature range in which the fit is valid.

	**$FA_{0.8}MA_{0.2}PbI_3$**				
Phase	Einstein osc.	A_i (meV)	$\hbar\omega_i$ (meV)	ZPC (meV)	T fitting range (K)
Cubic	Osc. ♯1	19(1)	6		>290
Tetra-II	Osc. ♯1	14(1)	6		180 - 250
Tetra-III	Osc. ♯1	7(1)	1.5	-50(20)	<125
	Osc. ♯2	-105(10)	14(2)		
	Osc. ♯1	7(1)	1.5	-55(20)	<180
	Osc. ♯2	-115(10)	15(2)		

For the tetragonal-III phase of $FA_{0.9}MA_{0.1}PbI_3$ the TE term function is a third-order polynomial $a_0 + a_1 T + a_2 T^2 + a_3 T^3$ with $a_0 = 0$, $a_1 = 5.51 \cdot 10^{-6}$ eV/K^2, $a_2 = -7.55 \cdot 10^{-8}$ eV/K^3, and $a_3 = 2.31 \cdot 10^{-10}$. Again, for the cubic and tetragonal-II phases a single Einstein oscillator is sufficient to describe the EP term, whereas for the low-temperature tetragonal-III phase the second Einstein oscillator corresponding to the anomalous electron-phonon coupling also has negative amplitude. The results of the least-square fitting to the first-derivative data points of Fig. 14 corresponding to the frequency and amplitude of the different Einstein oscillators are listed in Table S14.

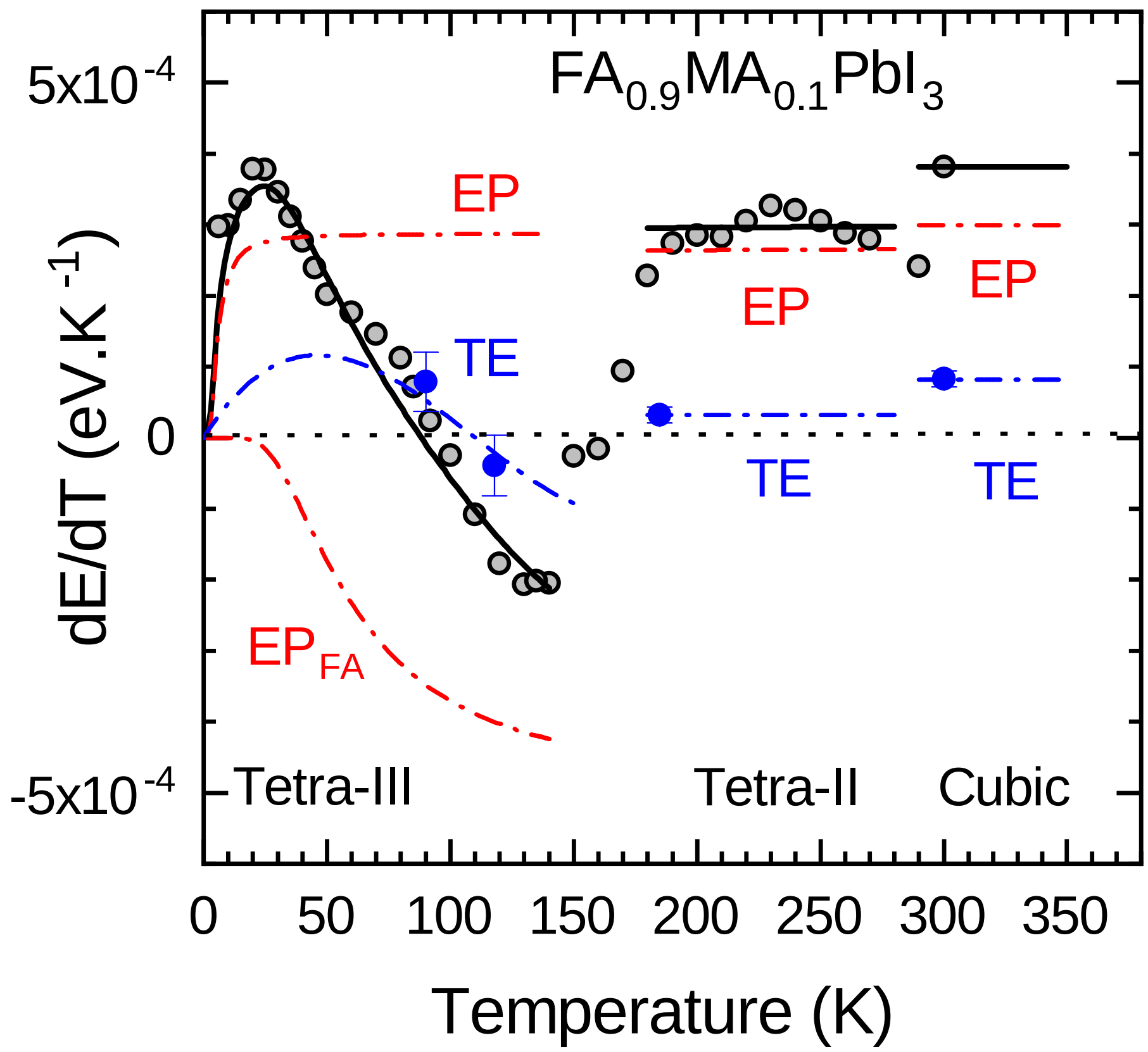

Figure S 14: Numerically calculated first derivative of the PL peak energy with respect to temperature (black-grey symbols) for $FA_{0.9}MA_{0.1}PbI_3$ mixed-cation perovskite. The solid black lines represent a fit only to the data points in the temperature range of stability of the different crystal phases, corresponding to the sum of the contribution of thermal expansion (TE, blue dot-dashed curve and symbols) and electron-phonon interaction (EP, red dot-dashed curve). The curve labeled EP_{FA} represents the additional Einstein oscillator introduced to account for the anomalous electron-phonon interaction due to coupling to FA rattler modes.

Table S 14: Einstein-oscillator parameters corresponding to the effective amplitude A_i and frequency ω_i describing the contribution from electron-phonon interaction $\left[\frac{\partial E_g}{\partial T}\right]_{EP}$ to the renormalization of the band gap in $FA_{0.9}MA_{0.1}PbI_3$. Numbers in parentheses are the error bars (uncertainty of the last digits) of the adjustable fitting parameters. Also listed are the value of the calculated zero-point corrections (ZPC) according to Eq. (8) and the temperature range in which the fit is valid.

	**$FA_{0.9}MA_{0.1}PbI_3$**				
Phase	Einstein osc.	A_i (meV)	$\hbar\omega_i$ (meV)	ZPC (meV)	T fitting range (K)
Cubic	Osc. $\sharp$1	21(1)	6		>300
Tetra-II	Osc. $\sharp$1	18.6(5)	6		180 - 280
Tetra-III	Osc. $\sharp$1	5(1)	1.5	-35(20)	<110
	Osc. $\sharp$2	-75(10)	15(2)		
	Osc. $\sharp$1	5(1)	1.5	-45(20)	<140
	Osc. $\sharp$2	-90(10)	16(2)		

According to Eq. (2), to evaluate the total gap temperature dependence (TE+EP terms) we have to calculate numerically the first derivative of the gap energies over temperature on a point-by-point basis. The black-grey symbols in Figs. S8 to S14 correspond to the first derivative data sets for the seven FA compositions measured. To obtain the EP term (or terms) for each composition by fitting, the analysis of the first-derivatives was performed piecewise within the temperature range of stability of the different phases, as indicated in the figures. The blue symbols and the blue dot-dashed curves correspond to the TE term resulting from Eq. (3) and their polynomial fits, respectively, using the parameters tabulated in Tables S1 to S7. The solid black curves in Figs. S8 to S14 represent the result of a least-squares fit to the first-derivative data points using the polynomial function describing the TE term (blue dot-dashed curve) plus the one or two Einstein-oscillator function (dashed red curve), as given by Eq. (7). The resulting amplitude and frequency of the Einstein oscillators are listed in Tables S8 to S14 for the corresponding FA contents and

plotted as a function of composition in Figs. S15a,b using closed red symbols and solid blue or blue-cyan symbols for Einstein oscillator corresponding to the normal and anomalous EP term, respectively.

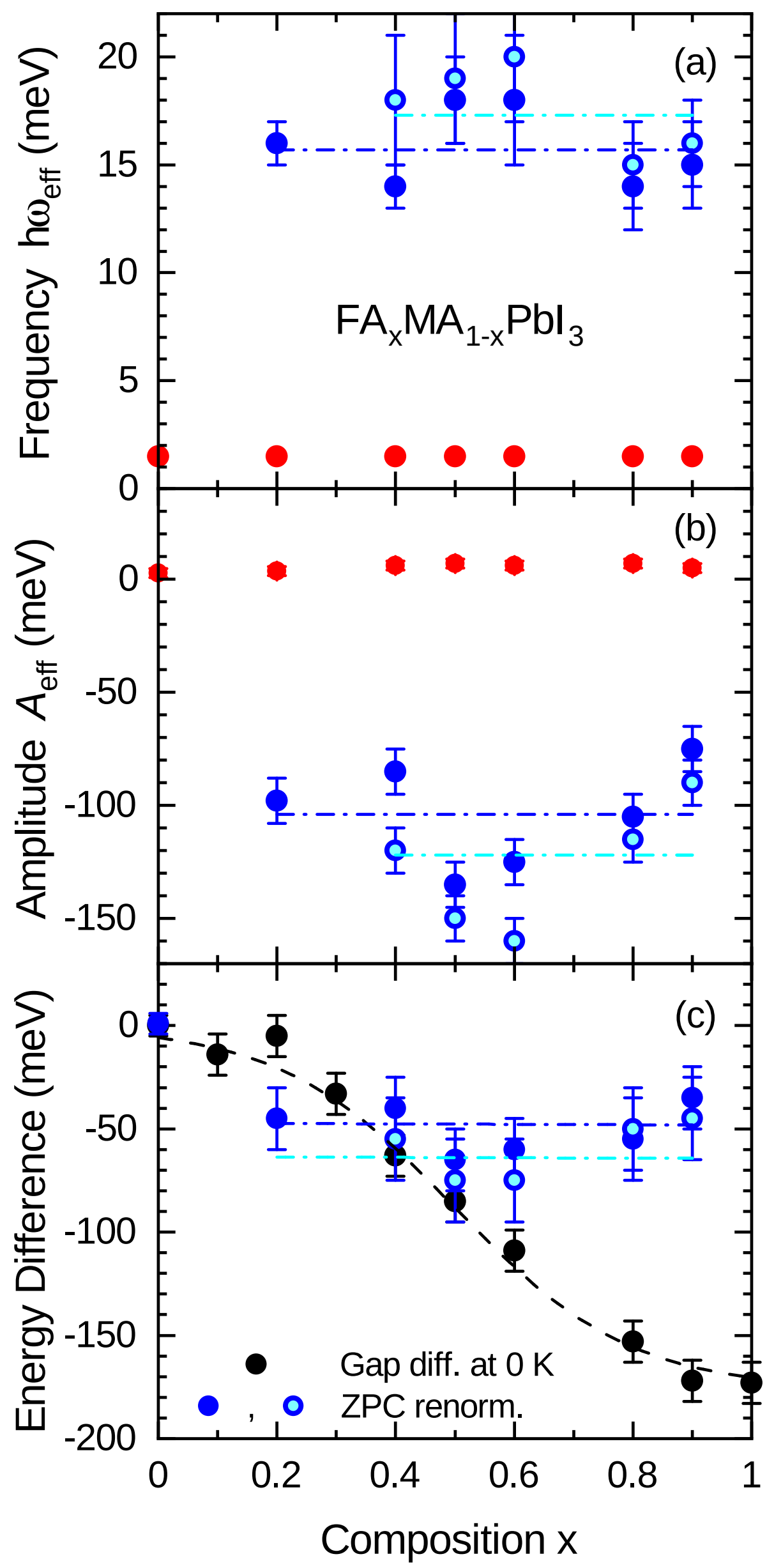

Figure S 15: **(a)** Frequency and **(b)** amplitude of the Einstein oscillator corresponding to the FA rattlers. **(c)** Differences between the gap energy extrapolated to 0 K for each composition referred to the gap of $MAPbI_3$ at 0 K plotted as a function of FA content (black symbols). Black dashed curve is a guide to the eye. Blue closed/open symbols represent the zero-point vibrations gap-corrections (ZPC) calculated using Eq. (8). Blue(cyan) dashed line represents linear fits to the closed(open) blue symbols, corresponding to the ZPC values obtained from fits performed in a shorter(wider) temperature range.

In Fig. S15c we have plotted the values of the energy difference between the gap extrapolated to 0 K for each FA composition, referred to that of $MAPbI_3$ (black symbols). The black dashed curve is a guide to the eye. Blue/blue-cyan closed symbols represent the zero-point vibrations gap corrections (ZPCs) calculated using Eq. (8). Blue(blue-cyan) dashed lines represents linear fits to the blue symbols, corresponding to the ZPC values obtained from fits performed in a shorter(wider) temperature range, as indicated in Tables S8 to S14. This comparison was made in view of the strong weight of the anomalous EP coupling in the total temperature renormalization of the gap, which made us think that it could also explain the strong composition dependence of the gap for the $FA_xMA_{1-x}PbI_3$ solid solutions. In fact, the gap energy extrapolated to 0 K (black symbols in Fig. S15c) decreases by ca. 170 meV when going from pure $MAPbI_3$ to $FAPbI_3$. We point out that this energy difference was calculated to be about 300 meV from DFT calculations including spin-orbit coupling[33] or 190 meV using the polymorphous model,[34] in excellent agreement with our experimental results. In contrast, the gap renormalization due to ZPC, though finite, it is essentially independent of FA content.

Note ♯6: Correlation between ferroelastic-domain formation & anomalous EP coupling

In Fig. S16a, we show the temperature dependence of the gap below 250 K for three very special cases, namely for FA contents of x =0, 0.1, and 0.2. The abrupt jump in gap energy signalizes the occurrence of the first-order phase transition from tetragonal (Tetra-I) to orthorhombic (Ortho). Above this transition temperature, all three compounds exhibit the same linear dependence of the gap with the typical positive slope characteristic of normal EP coupling with the phonons of the inorganic cage. However, in the Ortho phase, the temperature dependence of the gap differs in each case. For $MAPbI_3$ such dependence is non linear, though positive, and more importantly, it can be perfectly described using a single Einstein oscillator, representing the contribution from the normal EP term. In contrast, the sample with 20% FA content exhibits a pronounced bowing in

the gap temperature dependence, with a negative slope in the temperature range from 80 to 200 K. This phenomenon can only be reproduced by adding an oscillator with negative amplitude to account for the anomalous EP coupling to the FA-rattler modes.

The most interesting case is that of the 10% FA-content sample, which right after the transition into the Ortho phase exhibits a striking behavior of the gap (market with a red circle in Fig. S16a): On the one hand, there is a rigid blue-shift of five temperature data points with respect to the gap-vs-T curve at lower temperatures. On the other hand, these five points exhibit a negative slope which is exactly that of the sample with 20% FA. In Fig. S16b we reproduce the first derivative data of the sample with 10% FA content, but zooming into the temperature region of interest. We note that the precision of the measurements (scatter of the data points of the order of the symbol size) allows us to take these issues with the data points seriously. In fact, an inspection of the PL spectra of $FA_{0.1}MA_{0.9}PbI_3$ shown in Fig. S16c, corresponding to temperatures close to the Tetra-I to Ortho phase transition, tells us that the five spectra corresponding to the previously mentioned data points (wine-color curves) display a clear double-peak structure, which is indicative of the coexistence of both phases. The regions with tetragonal symmetry and larger volume exert tensile strain on the orthorhombic ones. This causes the rigid blue-shift of the gap, because its pressure coefficient is negative in this temperature range (see Table S1). The explanation for the negative temperature slope is even more enlightening. As shown in Fig. S16b, the overall tendency of the first derivative coincides very well with that of $MAPbI_3$, i.e., a gap temperature renormalization driven by thermal expansion and solely normal EP coupling (Ortho phase, blue-grey symbols in Fig. S16b). In contrast, the first derivative of these five temperature points is clearly negative (red symbols), being indicative of the contribution of the EP_{FA} term with negative sign. Although the precise mechanism is still unknown, we come to the important conclusion that the strain-driven formation of ferroelastic domains is instrumental for the activation of the anomalous EP_{FA} coupling term associated to the FA rattlers. These domains which are know to form in the Ortho phase after the phase transition,[35] are organized in stripes with the long tetragonal or orthorhombic axis, the preferential tilt axis, pointing at 90 deg. from stripe to stripe. Such an arrangement is the one

minimizing the macroscopic strain in the sample.

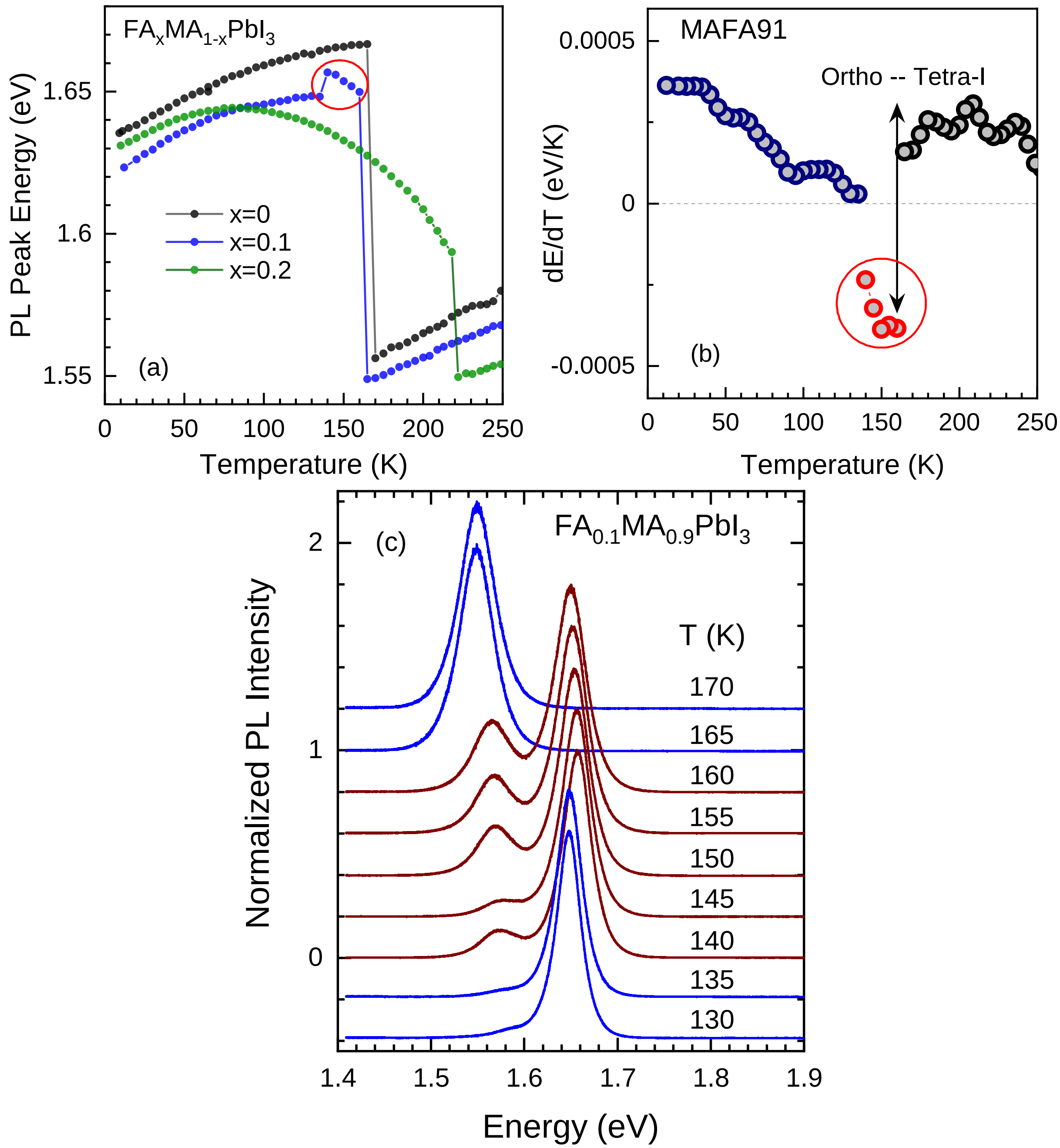

Figure S 16: **(a)** Maximum PL peak energy position plotted as a function of temperature, obtained from the PL line shape fits using a cross-product function (Eq. 1) for the first three compositions (x=0, 0.1, and 0.2) of the $FA_xMA_{1-x}PbI_3$ series. **(b)** First derivative of the PL peak energy E_0 with respect to temperature for $x = 0.1$, plotted as a function of temperature. **(c)** PL spectra of the $FA_{0.1}MA_{0.9}PbI_3$ in a small temperature range just across the Tetra-I to Ortho phase transition.

References

(1) Saidaminov, M. I.; Abdelhady, A. L.; Murali, B.; Alarousu, E.; Burlakov, V. M.; Peng, W.; Dursun, I.; Wang, L.; He, Y.; Maculan, G.; *et al.* High-Quality Bulk Hybrid Perovskite Single Crystals within Minutes by Inverse Temperature Crystallization. *Nature Commun.* **2015**, 6, 7586.

(2) Leguy, A. M. A.; Goñi, A. R.; Frost, J. M.; Skelton, J.; Brivio, F.; Rodríguez-Martínez, X.; Weber, O. J.; Pallipurath, A.; Alonso, M. I.; Campoy-Quiles, M.; Weller, M. T.; Nelson, J.; Walsh, A.; Barnes, P. R. F. Dynamic Disorder, Phonon Lifetimes, and the Assignment of Modes to the Vibrational Spectra of Methylammonium Lead Halide Perovskites. *Phys. Chem. Chem. Phys.* **2016**, 18, 27051-27066.

(3) Angel, R. J.; Bujak, M.; Zhao, J.; Gatta, G. D.; Jacobsen, S. D. Effective Hydrostatic Limits of Pressure Media for High-Pressure Crystallographic Studies, *J. Appl. Crystallogr.* **2007**, *40*, 26-32.

(4) Mao, H.-K.; Xu, J.; Bell, P. M. Calibration of the Ruby Pressure Gauge to 800 kbar under Quasi-Hydrostatic Conditions. *J. Geophys. Res.* **1986**, *91*, 4673-4676.

(5) Goñi, A. R.; Syassen, K. Optical properties of semiconductors under pressure. *Semicond. Semimetals* **1998**, 54, 247-425, and references therein.

(6) Ragan D. D.; Gustavsen, R.; Schiferl, D. Calibration of the Ruby R_1 and R_2 Fluorescence Shifts as a Function of Temperature from 0 to 600 K, *J. Appl. Phys.* **1992**, 72, 5539-5544.

(7) Francisco-López, A.; Charles, B. L.; Alonso, M. I.; Garriga, M.; Campoy-Quiles, M.; Weller, M. T.; Goñi, A. R. Phase Diagram of Methylammonium/Formamidinium Lead Iodide Perovskite Solid Solutions from Temperature-Dependent Photoluminescence and Raman Spectroscopies. *J. Phys. Chem. C* **2020**, *124*, 3448-3458.

(8) Francisco-López, A.; Charles, B.; Weber, O. J.; Alonso, M. I.; Garriga, M.; Campoy-Quiles, M.; Weller, M. T.; Goñi, A. R. Pressure-Induced Locking of Methylammonium Cations Versus Amorphization in Hybrid Lead Iodide Perovskites. *J. Phys. Chem. C* **2018**, 122, 22073-22082.

(9) D'Innocenzo, V.; Grancini, G.; Alcocer, M. J.-P.; Kandada, A. R. S.; Stranks, S. D.; Lee, M. M.; Lanzani, G.; Snaith, H. J.; Petrozza, A. Excitons Versus Free Charges in Organo-Lead Tri-Halide Perovskites. *Nature Commun.* **2014**, 5, 3486-3492.

(10) Wu, K.; Bera, A.; Ma, C.; Du, Y.; Yang, Y.; Li, L.; Wu, T. Temperature-Dependent Excitonic Photoluminescence of Hybrid Organometal Halide Perovskite Films. *Phys. Chem. Chem. Phys.* **2014**, 16, 22476-22481.

(11) Dong, Q.; Fang, Y.; Shao, Y.; Mulligan, P.; Qiu, J.; Cao, L.; Huang, J. Electron-Hole Diffusion Lengths >175 μm in Solution-Grown $CH_3NH_3PbI_3$ Single Crystals. *Sci.* **2015**, 347, 967-970.

(12) Galkowski, K.; Mitioglu, A. A.; Surrente, A.; Yang, Z.; Maude, D. K.; Kossaki, P.; Eperon, G. E.; Wang, J. T.-W.; Snaith, H. J.; Plochocka, P.; et al. Spatially Resolved Studies of the Phases and Morphology of Methylammonium and Formamidinium Lead Tri-Halide Perovskites. *Nanoscale* **2017**, 9, 3222-3230.

(13) Wojdyr, M. Fityk: A General-Purpose Peak Fitting Program. *J. Appl. Crystallogr.* **2010**, 43, 1126-1128.

(14) Francisco-López, A.; Charles, B. L.; Alonso, M. I.; Garriga, M.; Weller, M. T.; Goñi, A. R. Photoluminescence of Bound-Exciton Complexes and Assignment to Shallow Defects in Methylammonium/Formamidinium Lead Iodide Mixed Crystals. *Adv. Optical Mater.* **2021**, 2001969/1-9.

(15) Fasahat, S.; Fiuza-Maneiro, N.; Schäfer, B.; Xu, K.; Gómez-Graña, S.; Alonso, M. I.; Polavarapu, L.; Goñi, A. R. Sign of the gap temperature dependence in $CsPb(Br,Cl)_3$

nanocrystals determined by Cs-rattler-mediated electron-phonon coupling, *J. Phys. Chem. Lett.* **2025**, 16, 1134-1141.

(16) Lautenschlager, P.; Allen, P. B.; Cardona, M. Temperature Dependence of Band Gaps in Si and Ge. *Phys. Rev. B* **1985**, 31, 2163-2171.

(17) Gopalan, S.; Lautenschlager, P.; Cardona, M. Temperature Dependence of the Shifts and Broadenings of the Critical Points in GaAs. *Phys. Rev. B* **1987**, 35, 5577-5584.

(18) Göbel, A.; Ruf, T.; Cardona, M.; Lin, C. T.; Wrzesinski, J.; Steube, M.; Reimann, K.; Merle, J.-C.; Joucla, M. Effects of the isotopic composition on the fundamental gap of CuCl. *Phys. Rev. B* **1998**, 57, 15183-15190.

(19) Cardona, M. Electron-Phonon Interaction in Tetrahedral Semiconductors. *Solid State Commun.* **2005**, 133, 3-18.

(20) Fasahat, S.; Schäfer, B.; Xu, K.; Fiuza-Maneiro, N.; Gómez-Graña, S.; Alonso, M. I.; Polavarapu, L.; Goñi, A. R. Absence of Anomalous Electron-Phonon Coupling in the Temperature Renormalization of the Gap of $CsPbBr_3$ Nanocrystals. *J. Phys. Chem. C* **2025**, 129, 453-463.

(21) Jacobsson, T. J.; Schwan, L. J.; Ottosson, M.; Hagfeldt, A.; Edvinsson, T. Determination of Thermal Expansion Coefficients and Locating the Temperature-Induced Phase Transition in Methylammonium Lead Perovskites Using X-ray Diffraction, *Inorg. Chem.* **2015**, 54, 10678-10685.

(22) Whitfield, P. S.; Herron, N.; Guise, W. E.; Page, K.; Cheng, Y. Q.; Milas, I.; Crawford, M. K. Corrigendum: Structures, Phase Transitions and Tricritical Behavior of the Hybrid Perovskite Methyl Ammonium Lead Iodide, *Sci. Rep.* **2017**, 7, 42831.

(23) Whitfield, P. S.; Herron, N.; Guise, W. E.; Page, K.; Cheng, Y. Q.; Milas, I.; Crawford, M.

K. Structures, Phase Transitions and Tricritical Behavior of the Hybrid Perovskite Methyl Ammonium Lead Iodide, *Sci. Rep.* **2016**, 6, 35685/1-15.

(24) Weber, O. J.; Charles, B. L.; Weller, M. T. Phase Behaviour and Composition in the Formamidinium-Methylammonium Hybrid Lead Iodide Perovskite Solid Solution, *J. Mater. Chem. A* **2016**, 4, 15375-15382.

(25) Fabini, D. H.; Stoumpos, C. C.; Laurita, G.; Kaltzoglou, A.; Kontos, A. G.; Falaras, P.; Kanatzidis, M. G.; Seshadri, R. Reentrant Structural and Optical Properties and Large Positive Thermal Expansion in Perovskite Formamidinium Lead Iodide, *Angew. Chem.* **2016**, 128, 15618-15622.

(26) Cordero, F.; Craciun, F.; Trequattrini, F.; Generosi, A.; Paci, B.; Paoletti, A. M.; Pennesi, G. Stability of Cubic $FAPbI_3$ from X-ray Diffraction, Anelastic, and Dielectric Measurements, *J. Phys. Chem. Lett.* **2019**, 10, 2463-2469.

(27) Jaffe, A.; Lin, Y.; Beavers, C. M.; Voss, J.; Mao, W. L.; Karunadasa, H. I. High-Pressure Single-Crystal Structures of 3D Lead-Halide Hybrid Perovskites and Pressure Effects on their Electronic and Optical Properties. *ACS Cent. Sci.* **2016**, 2, 201-209.

(28) Szafrański, M.; Katrusiak, A. Mechanism of Pressure-Induced Phase Transitions, Amorphization, and Absorption-Edge Shift in Photovoltaic Methylammonium Lead Iodide. *J. Phys. Chem. Lett.* **2016**, 7, 3458-3466.

(29) Pieniazek, A.; Dybala, F.; Polak, M. P.; Przypis, L.; Herman, A. P.; Kopaczek, J.; Kudrawiec, R. Bandgap Pressure Coefficient of a $CH_3NH_3PbI_3$ Thin Film Perovskite, *J. Phys. Chem. Lett.* **2023**, 14, 6470-6476.

(30) Serrano, J.; Schweitzer, Ch.; Lin, C. T.; Reimann, K.; Cardona, M.; Fröhlich, D. Electron-Phonon Renormalization of the Absorption Edge of the Cuprous Halides. *Phys. Rev. B* **2002**, 65, 125110/1-7.

(31) Bhosale, J.; Ramdas, A. K.; Burger, A.; Muñoz, A.; Romero, A. H.; Cardona, M.; Lauck, R.; Kremer, R. K. Temperature Dependence of Band Gaps in Semiconductors: Electron-Phonon Interaction. *Phys. Rev. B* **2012**, 86, 195208/1-10.

(32) Francisco López, A.; Charles, B.; Weber, O. J.; Alonso, M. I.; Garriga, M.; Campoy-Quiles, M.; Weller, M. T.; Goñi, A. R. Equal Footing of Thermal Expansion and Electron-Phonon Interaction in the Temperature Dependence of Lead Halide Perovskite Band Gaps. *J. Phys. Chem. Lett.* **2019**, 10, 2971-2977.

(33) Senno, M.; Tinte, S. Mixed Formamidinium-Methylammonium Lead Iodide Perovskite from First-Principles: Hydrogen Bonding Impact on the Electronic Properties. *Phys. Chem. Chem. Phys.* **2021**, 23, 7376-7385.

(34) Zacharias, M.; Volonakis, G.; Pedesseau, L.; Katan, C.; Giustino, F.; Even, J. Roadmap for Electronic Structure, Anharmonicity, and Electron-Phonon Calculations in Locally Disordered Inorganic and Hybrid Halide Perovskites. *Phys. Rev. B* **2026**, 113, 085118/1-45.

(35) Ambrosio, F.; De Angelis, F.; Goñi, A. R. The Ferroelectric-Ferroelastic Debate about Metal Halide Perovskites. *J. Phys. Chem. Lett.* **2022**, *13*, 7731-7740.
